# CREB3L1 and CREB3L2 control Golgi remodelling during decidualization of endometrial stromal cells

**DOI:** 10.3389/fcell.2022.986997

**Published:** 2022-10-13

**Authors:** Daniele Pittari, Marco Dalla Torre, Elena Borini, Barbara Hummel, Ritwick Sawarkar, Claudia Semino, Eelco van Anken, Paola Panina-Bordignon, Roberto Sitia, Tiziana Anelli

**Affiliations:** ^1^ Faculty of Medicine, Vita-Salute San Raffaele University, Milan, Italy; ^2^ Division of Genetics and Cell Biology, IRCCS Ospedale San Raffaele, Milan, Italy; ^3^ Max Planck Institute of Immunobiology and Epigenetics, Freiburg, Germany; ^4^ Medical Research Council (MRC), University of Cambridge, Cambridge, United Kingdom; ^5^ Division of Neuroscience, IRCCS Ospedale San Raffaele, Milan, Italy

**Keywords:** secretory pathway, vesicular transport, differentiation, transcriptomics analysis, collagen

## Abstract

Upon progesterone stimulation, Endometrial Stromal Cells (EnSCs) undergo a differentiation program into secretory cells (decidualization) to release in abundance factors crucial for embryo implantation. We previously demonstrated that decidualization requires massive reshaping of the secretory pathway and, in particular, of the Golgi complex. To decipher the underlying mechanisms, we performed a time-course transcriptomic analysis of *in vitro* decidualizing EnSC. Pathway analysis shows that Gene Ontology terms associated with vesicular trafficking and early secretory pathway compartments are the most represented among those enriched for upregulated genes. Among these, we identified a cluster of co-regulated genes that share CREB3L1 and CREB3L2 binding elements in their promoter regions. Indeed, both CREB3L1 and CREB3L2 transcription factors are up-regulated during decidualization. Simultaneous downregulation of CREB3L1 and CREB3L2 impairs Golgi enlargement, and causes dramatic changes in decidualizing EnSC, including Golgi fragmentation, collagen accumulation in dilated Endoplasmic Reticulum cisternae, and overall decreased protein secretion. Thus, both CREB3L1 and CREB3L2 are required for Golgi reshaping and efficient protein secretion, and, as such, for successful decidualization.

## Introduction

With only few exceptions, secretory proteins are cotranslationally translocated into the Endoplasmic Reticulum (ER), where they undergo oxidative folding, N-glycosylation and other posttranslational modifications under the control of devoted chaperones and enzymes (Braakman and Bulleid, 2011). Protein quality control (QC) mechanisms ensure that only native proteins proceed along the secretory pathway (Anelli and Sitia, 2008; Sun and Brodsky, 2019); proteins that fail to fold are instead destined for degradation *via* cytosolic proteasomes (Ruggiano et al., 2014; Christianson and Carvalho, 2022). Cargo receptors (such as ERGIC-53) are active at ER exit sites (ERES) to accelerate the secretion of folded molecules (Zhang, 2009; Anelli and Panina-Bordignon, 2019). Recruiting cytosolic COPII components, they ensure efficient movement of secretory proteins from the ER to the Golgi. In the Golgi compartment, cargo proteins are further processed and finally get secreted or exposed on the plasma membrane (Schwabl and Teis, 2022). To maintain membrane homeostasis and organelle composition, anterograde traffic is paralleled by COPI-dependent retrograde transport. All these events must be precisely coordinated.

The organelles of the secretory compartment are particularly developed in professional secretors, since these are the sites devoted to the production of almost all proteins destined to be secreted (Federovitch et al., 2005). Indeed, their components are selectively up-regulated in all well-studied differentiation programs towards secretory phenotypes (Harding et al., 2001; Christis et al., 2010). This is the case, for example, of B to plasma cell differentiation: after the encounter with antigens, B lymphocytes first proliferate and then transform into *protein factories*, dramatically enlarging their ER under the control of Ire1, Xbp1 and other Unfolded Protein Response (UPR)-related factors (Reimold et al., 2001; Iwakoshi et al., 2003; Van Anken et al., 2003).

**FIGURE 1:**
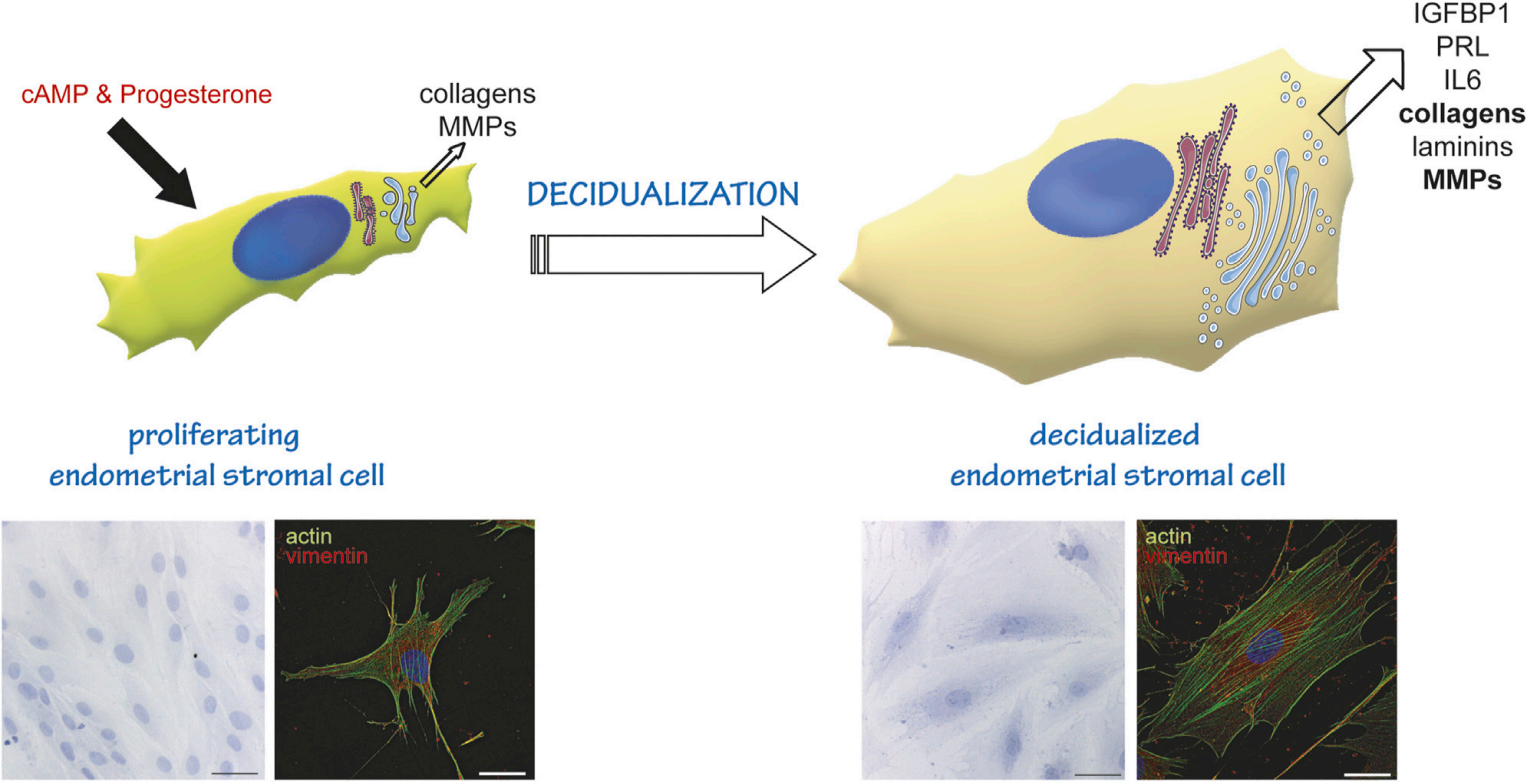
Endometrial stromal cells as a model of cell differentiation to a secretory phenotype. In the proliferative state, EnSC show a fibroblastoid shape, and produce and secrete some collagens and metalloproteases (MMPs). Decidualisation (induced *in vitro* by cAMP and Progesterone stimulation) induces a reshaping of the entire cell and of the secretory compartment. Cells stop proliferating and enlarge, acquiring an epithelioid-like shape. They enlarge their ER and Golgi compartment (Anelli et al., 2021) and transform into professional secretors, expressing and secreting decidualization markers (IGFBP1, PRL, IL6), more and different collagens, MMPs and laminins. The lower insets on the left show a Hematoxylin-Eosin staining of cells at day 0 (proliferating, left) and day 6 of decidualization (right). Images were acquired with a ×20 objective; bar: 60 µm. The lower insets on the right show an immunofluorescence of proliferating (left) and decidualized cells (right) stained with fluorescently labelled Phalloidin (actin, green) and anti-vimentin antibody (red). Note the change in cytoskeletal reorganization following stimulation. Images were acquired with a ×60 objective; bar: 20 µm.

Another example of a secretory differentiation program is the decidualization of Endometrial Stromal Cells (EnSC) (Figure 1) (Gellersen and Brosens, 2014). With each menstrual cycle, human EnSCs undergo a phase of intense proliferation. Then, upon the post-ovulatory increase of progesterone, cells exit the cell cycle, enlarge and differentiate into secretory cells that produce different mediators (IL-6, Tissue Factor, IGFBP1, Matrix metalloproteinases (MMPs), collagens, Prolactin) to anticipate blastocyst implantation, should fertilization occur. We recently demonstrated that decidualization entails massive rearrangements of EnSCs cytoarchitecture, with enlargement of the entire cell, and most prominently of the secretory pathway organelles (Anelli et al., 2021). In contrast to what occurs during B to plasma cell differentiation, however, in decidualizing EnSCs the Golgi complex expands more than the ER (Anelli et al., 2021). This divergence in how the secretory pathway differentiates might be due to the different cargoes produced by either cell type. The more pronounced Golgi enlargement, that is also a hallmark of decidualization, may be required to sustain bulk production of highly O-glycosylated proteins (Noske et al., 2008).

How are the above morphological rearrangements orchestrated during decidualization? We previously showed (Anelli et al., 2021) that decidualization entails minimal activation of the UPR pathways that are instead key in plasma cell development. Quiescent B lymphocytes secrete little if any proteins: hence, they need to build up a secretory machinery almost from scratch, which explains the reliance of B to plasma cell differentiation on the UPR. Conversely, EnSCs secrete proteins also before progesterone stimulation. Therefore, they apparently modulate the rates of protein production and transport rather than massively expanding the ER during decidualization.

However, it is not clear whether the morphological changes of decidualizing EnSCs are inherently encoded in the transcriptional program of decidualization itself or, *vice versa*, they are driven by the increased cargo production and secretion.

This study aims to elucidate the mechanisms that orchestrate cellular reshaping during decidualization: we focused on the characterization of the gene expression trends and on the prediction and experimental validation of transcription factors implicated in the enlargement of the Golgi compartment. To this end, we performed unbiased transcriptomic analyses of *in vitro* decidualization of human immortalized endometrial stromal cells (T-HESC) at different time points after progesterone stimulation. A distinct upregulation of the ER, Golgi and lysosomal proteins is evident at the (relative) expense of the cytosolic components. The members of a cluster of upregulated transcripts related to the early secretory compartment share binding sites for CREB3L1 and CREB3L2. Accordingly, both transcription factors are not only upregulated during decidualization, but also necessary for Golgi remodelling. Their ablation has dramatic consequences on protein secretion, with collagen accumulating in dilated ER cisternae, which abrogates the efficient decidualization process. Our results demonstrate that the activation of CREB3L1 and CREB3L2 is key for the reshaping of the early secretory compartment during decidualization and for achieving a secretory phenotype.

## Results

### *In vitro* decidualization of T-HESC entails extensive transcriptomics rearrangements

To characterize the gene expression changes occurring in decidualization, we performed bulk RNA sequencing of human immortalized endometrial stromal cells (T-HESC) under basal conditions (0 h) and at various time points during *in vitro* decidualization (i.e. 6 h, 18 h, day 1, 1.5, 2, 3, and 6 after hormonal stimulation). We focused on short intervals between early time points to better discriminate transcriptional modifications characterizing the first phases of decidualization.

**FIGURE 2:**
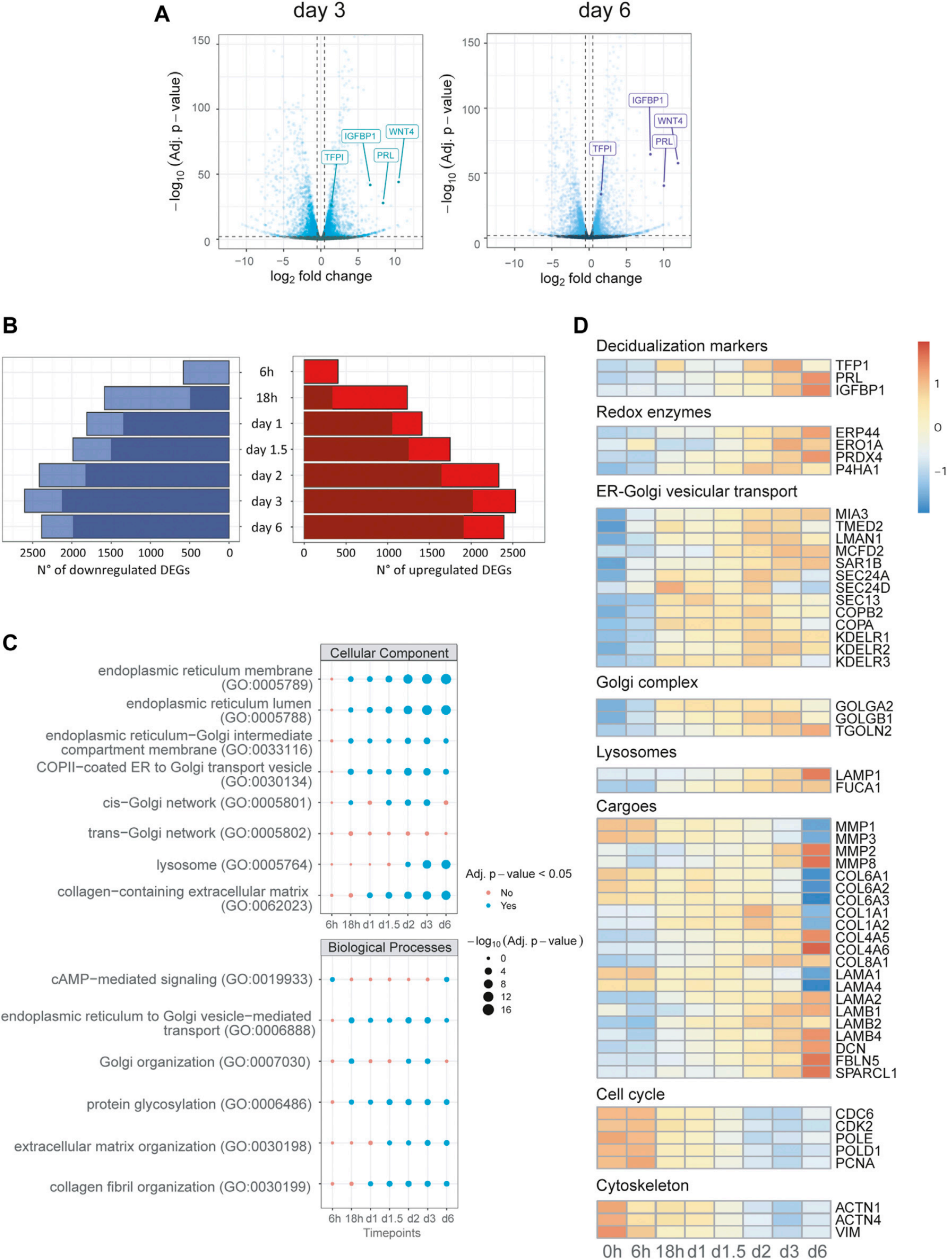
Transcriptional reprogramming of the secretory compartment is an early event in decidualization. Differential expression analysis and Over-Representation Analysis (ORA) of time course bulk RNA-seq. **(A)** Volcano plots of day 3 and 6, gene labelling for decidualization markers (PRL, IGFBP1, TFP1), which act as positive controls for our experimental set up. **(B)** Number of DEGs called for each timepoint grouped for upregulation or downregulation: for each bar, the darker shades of colour represent the number of genes called DEGs also at the previous time point. **(C)** ORA of upregulated DEGs for GO terms for each timepoint: early enrichment is observed for pathways related to the compartments and biological processes of the secretory pathway. **(D)** Heatmap of representative DEGs for canonical biological functions involved in decidualization up to day 6 of differentiation. Note the upregulation of genes of the early secretory pathway and the downregulation of genes correlated with cell cycle and DNA repair. The rearrangements of cargos (collagens, laminins) is already evident just a few hours after stimulation.

Differential expression analysis (DEA) was performed by pairwise comparisons of samples from each time point with untreated samples. The volcano plots comparing expression patterns at day 3 and day 6 with basal expression (Figure 2A) highlight the upregulation of known markers [Prolactin (PRL), WNT4, IGFBP1 and TFBI], as a positive control of ongoing decidualization. Our results show that *in vitro* decidualization is accompanied by a profound transcriptome rearrangement, featuring up or down-regulation of thousands of genes. As differentiation progresses, the number of differentially expressed genes (DEGs) progressively increases: from 984 at 6 h, their number rises to its maximum of 5,136 DEGs at day 3. At the end of our differentiation protocol (day 6), the number of DEGs is 4,780 (Figure 2B, Supplementary Table S1).

### Transcriptional profile of stimulated T-HESC reflects physiological decidualization program

To get insights into the biological significance of the various DEGs observed at each time point, we performed over-representation analysis (ORA) for the major Gene Ontology annotations: biological processes (BP), cellular component (CC), and molecular function (MF). As expected, ORA reveals significant enrichment at 6 h for terms related to response to hormonal stimulation (cAMP-mediated signalling, glucocorticoid receptor binding, 3′,5′-cyclic-AMP phosphodiesterase activity; Supplementary Table S2A–E, Figure 2C). An enrichment for terms associated with ER-Golgi vesicular trafficking, protein glycosylation and Golgi organization follows at the next time point (18 h). These terms turn out to be predominant at all further time points, suggesting the centrality of secretory pathway reshaping among upregulated DEGs from the early to last stages of our decidualization protocol. In accordance, we found the same trends for terms associated with the main compartments of the early secretory pathway, such as ER lumen and membranes, ER-Golgi intermediate compartment (ERGIC), cis-Golgi and lysosomes (Figure 2C, Supplementary Table S2C).

Conversely, downregulated DEGs show enrichment for terms associated with DNA replication, DNA repair, cell cycle progression, and nuclear compartment (Supplementary Table S2B–D). As observed for upregulated genes, the enrichment for these terms was consistent throughout the decidualization process from 18 h onwards. In addition, an early enrichment for terms related to cytoskeleton, focal adhesion and cell-cell junctions is seen at 6 h (Figure 2C; Supplementary Table S2C).

The expression profile of representative genes of the main compartments or biological processes is shown in Figure 2D. The panel indicates a coordinated upregulation of genes encoding proteins involved in disulfide bond formation in the ER (redox enzymes) and of genes of ER to Golgi vesicular trafficking and of the Golgi complex. As expected, the expression of genes involved in cell cycle progression is downregulated, as well as cytoskeleton genes. The analysis also reflects a shift in the cargo produced during the decidualization process (Carbone et al., 2006; Shi et al., 2020): while proliferating EnSCs produce Collagen type VI, after decidualization they downregulate collagen type VI and upregulate instead collagen type I, IV, and VIII. Changes occurs also in the types of laminins expressed.

To further corroborate our results, we compared the DEGs identified at the end of the decidualization protocol (day 6) with a list of 115 gene products experimentally validated for showing functional reprogramming upon decidualization of human endometrial stromal cells (Gellersen and Brosens, 2014). Indeed, we found an almost perfect overlap between this list and prominent upregulated DEGs identified by our analysis (Supplementary Table S3), further supporting our choice for *in vitro* differentiation of T-HESC as a model to study the decidualization process.

### Time-series clustering reveals a class of co-regulated genes linked with vesicular transport

**FIGURE 3:**
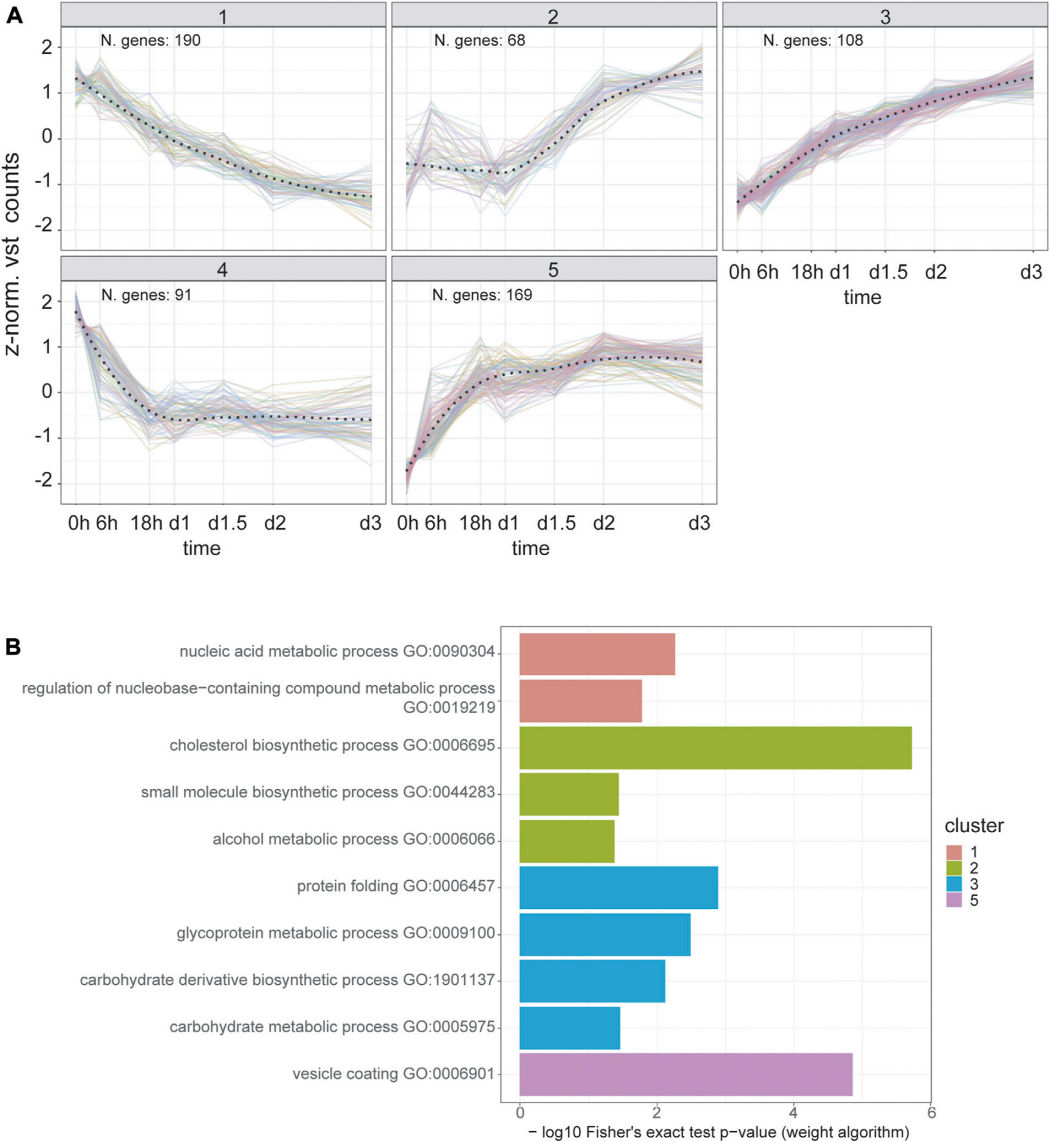
Time series clustering dissects the temporal orchestration of ER and Golgi apparatus DEGs. **(A)** Temporal expression trends for DEGs annotated to “Golgi Apparatus” and/or “Endoplasmic Reticulum” GO cellular component terms. *Y*-axis represents the z-normalized, averaged expression value transformed using DESEQ2 variance stabilized transformation. The overall trends for each cluster (dashed line) were estimated using a LOESS fit. **(B)** Over-representation analysis results for biological process GO terms in each cluster. Fisher’s exact test was carried out using the weight algorithm proposed in Alexa et al., 2006, implemented in TopGO. Cluster five shows enrichment for vesicular transport terms, reflecting the presence of the main components of COPI, COPII and cargo receptors genes (Supplementary Table 4). Cluster four does not show terms with a significant enrichment (i.e. *p* value < 0.05).

To predict shared cis-regulatory elements orchestrating early secretory pathway reshaping and, possibly, Golgi expansion, we used a data-driven approach that takes advantage of our bulk RNA-seq time-course design. First, we created a subset of 626 DEGs by selecting those annotated in GO terms for the cellular component (CC) related to the ER and Golgi apparatus, as well as called differentially expressed in the first 3 days of differentiation. Then, we performed partitional clustering based on their co-expression patterns. In this way, we identified five different clusters, two describing downregulation and three describing upregulation trends (Figure 3A). In cluster 5, one of those describing upregulation trends, we identified the presence of canonical genes involved in vesicular transport, such as COPI and COPII components, and cargo receptors (SEC23A, SEC24A, SEC16A, COPZ1/2, COPA, COPB2, LMAN1, MIA3; Figure 3A; Supplementary Table S4). ORA for biological processes (BP) GO terms also reveals functional enrichment for vesicular transport genes in this cluster (Figure 3B). Next, we predicted common cis-regulatory elements in each cluster using the tool RcisTarget. Interestingly, cluster five scores the highest enrichment values for top putative transcription factors associated with vesicular transport promotion, such as CREB3L2, CREB3L1, CREB3, ATF6, and Xbp1 (Supplementary Table S5).

### CREB3L1 and CREB3L2 are upregulated during decidualization

**FIGURE 4:**
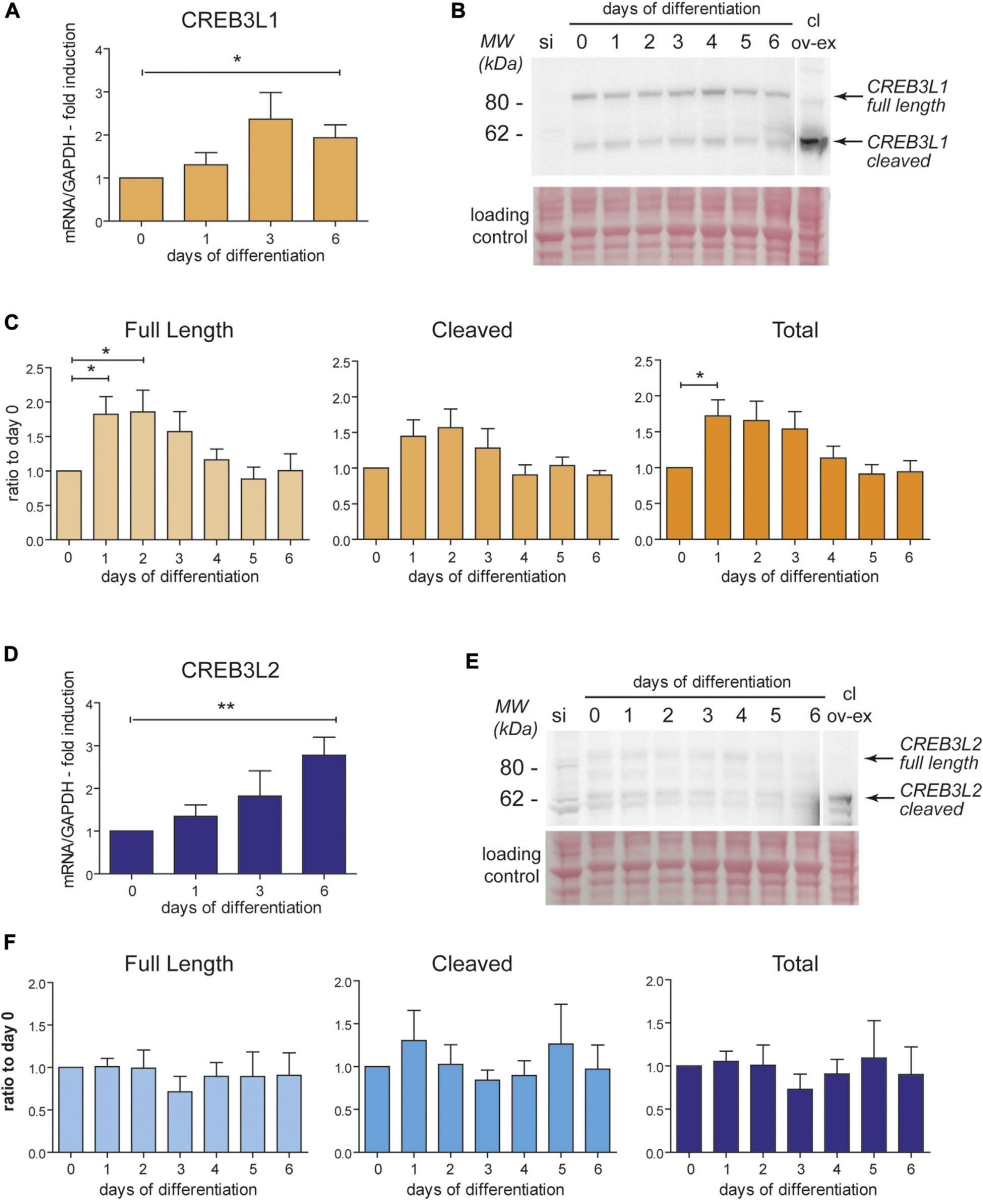
CREB3L1 and two are upregulated during decidualization. T-HESC were treated with cAMP and progesterone and samples (RNA and total protein lysates) were collected every day up to day6 after the treatment. **(A)** mRNA levels of CREB3L1 were quantified using qPCR, normalized on GAPDH and expressed as fold induction on day 0. The mRNA increases after stimulation up to day 3 and then start decreasing. Average of samples from six independent experiments ± SEM. *p < 0.05, *t*-test vs day0. **(B)** 40 µg of total proteins were analyzed in SDS-PAGE, under reducing conditions on a 4–12% precasted acrylamide gel. As a negative control, a sample of cells subjected to CREB3L1 siRNA the day before were loaded (si). As a positive control to discriminate the cleaved form, 40 µg of protein extract of HeLa cells transiently transfected with cleaved CREB3L1 was loaded (cl ov-ex). The full length and the cleaved forms are indicated by arrows on the right. The ponceau staining of the nitrocellulose is shown as a loading control. The lane showing the over-expressed cleaved form comes from a lower exposure image of the same nitrocellulose. **(C)** Densitometric quantification of six independent experiments as the one showed in B (average ± SEM). An increase at the protein levels in the first days parallels the increase in the mRNA level shown in panel A. *p < 0.05, *t*-test vs day 0. **(D)** mRNA levels of CREB3L2 were analyzed in qPCR. The mRNA increases after stimulation up to day 6. Average of samples from six independent experiments ± SEM. *p < 0.05, *t*-test vs day 0. **(E)** 40 µg of total proteins were analyzed as described in panel B. As a negative control, a sample of cells subjected to CREB3L2 siRNA the day before were loaded (si). As a positive control to visualized the cleaved form, 40ug of protein extract of HeLa cells transiently transfected with cleaved CREB3L2 was loaded (cl ov-ex). The lane showing the over-expressed cleaved form comes from a lower exposure image of the same nitrocellulose. The full length and the cleaved forms are indicated by arrows on the right. The ponceau staining of the nitrocellulose is shown as a loading control. **(F)** Densitometric quantification of five independent experiments as the one showed in B (average ± SEM). Despite the continuous increase of the mRNA of CREB3L2 during decidualization, nothing seems to change at the protein level.

Since our previous studies identified only a minimal induction of ATF6, and no activation of the Xbp1 pathway during decidualization (Anelli et al., 2021), we focused our attention on CREB3L1 and CREB3L2, two transcription factors (TF) that have been previously linked to collagen secretion and vesicular trafficking in other cell types (Fox et al., 2010; Asada et al., 2011; Chan et al., 2011; Ishikawa et al., 2017; Khetchoumian et al., 2019; Jhonson et al., 2020). These transcription factors are under basal conditions localized in the ER, where they are readily ubiquitinated, extracted from the membrane and degraded (Kondo et al., 2012). Upon activation, CREB3L1 and CREB3L2 translocate to the Golgi complex, where they undergo RIP (Regulated Intramembrane Proteolysis) by the proteases S1P and S2P. This proteolytic cleavage event releases their cytosolic domains that migrate to the nucleus to exert their function as TFs (Kondo et al., 2007; Murakami et al., 2009). qPCR shows that, during *in vitro* decidualization, both CREB3L1 and CREB3L2 are upregulated at the mRNA level, although with slightly different kinetics (Figure 4): the mRNA of CREB3L1 shows a trend with a peak at day 3 (Figure 4A), while CREB3L2 increases steadily throughout the decidualization process, up till day 6 (Figure 4D). At the protein level, CREB3L1 is already upregulated on day 1 of decidualization, and then its levels start to decrease (Figures 4B,C). Cleaved CREB3L1 seems to peak at day 1 and then decreases. On the contrary, we did not detect any induction of CREB3L2 at the protein level (Figures 4E,F), despite its mRNA increases during decidualization. We could hypothesise a fast turnover of this protein, preventing its accumulation, but this phenomenon awaits further clarification.

### CREB3L1 and CREB3L2 are needed for efficient decidualization

**FIGURE 5:**
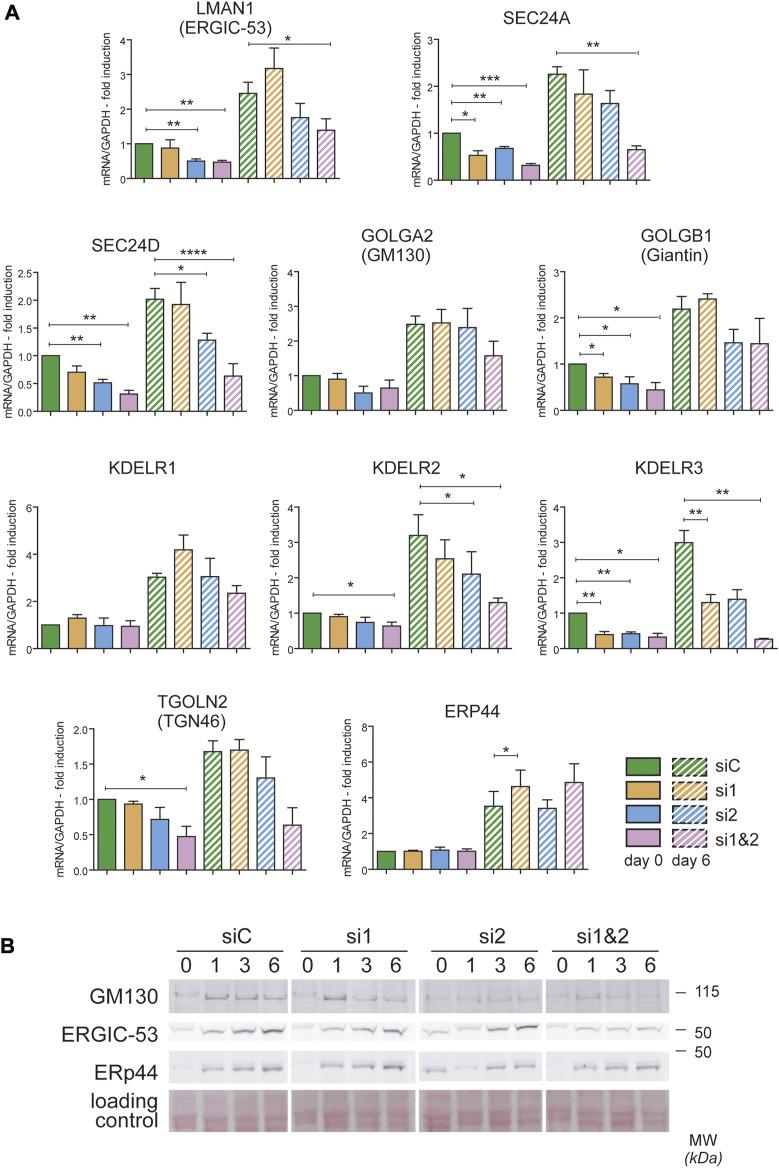
CREB3L1 and CREB3L2 drive the upregulation of many genes of the early secretory pathway during decidualization. T-HESC were treated with scramble duplexes (siC, green bars), with duplexes specific for CREB3L1 (si1, orange), CREB3L2 (si2, light blue) or both (si1&2, pink). The day after treatment (day 0) cells were collected or treated with cAMP and progesterone and then collected at day 6 of decidualization. **(A)** mRNA for different genes encoding proteins of the early secretory pathway were analyzed by qPCR and expressed as fold induction to day 0 siC after normalization on GAPDH. Solid bars represent values at day 0, striped bars values at day 6. Average of 3–4 independent experiments ± SEM. *p < 0.05; **p < 0.01; ***p < 0.001; ****p < 0.0001, *t*-test vs day 0 siC. The KD of CREB3L1 and/or 2 affects the expression of the majority of the genes analyzed, both at day 0 and after 6 days of decidualization. **(B)** Total protein extracts were loaded on SDS PAGE under reducing conditions (40 µg of proteins/lane). The behavior of GM130, ERGIC-53 and ERp44 are shown. While ERGIC-53 and GM130 suffer from the absence of CREB3L1 and/or 2, the protein levels of ERp44 are unaffected. A part of the ponceau is shown as a loading control.

To gain insight into the role of CREB3L1 and 2 during decidualization, we used small interfering RNAs targeted to CREB3L1 and 2, individually or in combination. Silencing was started before inducing decidualization (Day -1) so that, when decidualization was induced (Day 0), expression of CREB3L1 and 2 was already suppressed (Supplementary Figures 1A and 4B,E). Silencing was repeated after 3 days, to sustain the downregulation of the transcription factors for the entire experiment (see Material and Methods section and Supplementary Figure 1A). qPCR analysis (Supplementary Figure 1A) indicates that no major compensatory responses (up-regulation of one TF in the absence of the other) are activated during the experiments. Cells responded as expected to hormonal stimulation also upon CREB3L1 and/or 2 silencing, as shown by the increase in PRL mRNA levels (Supplementary Figure 1B). However, suppression of both transcription factors dramatically impaired the expression of ERGIC-53 (LMAN1), SEC24A and SEC24D (involved in ER to Golgi vesicular traffic), and of KDELR2 and 3 (involved in retrieving ER chaperone escapees from the Golgi). Trends suggesting minor effects were observed for other Golgi-related genes, such as GOLGA2 (GM130), giantin (GOLGB1) and TGN46 (TGOLN2) (Figure 5A). Moreover, for most of the transcripts analysed, a strong effect of CREB3L1/2 silencing was observed already at day 0, which suggests a major role of the two transcription factors in regulating their expression also in resting conditions. We excluded a nonspecific effect of the silencing procedure, since other secretory pathway genes (such as ERp44 and KDELR1) were not affected.

The effects of CREB3L1/2 silencing were confirmed at the protein level (Figure 5B). A reduced induction of both ERGIC-53 and GM130 was observed by western blotting, especially upon silencing of both transcription factors. As expected from the mRNA analysis, no effect on ERp44 induction was observed.

### Ablation of CREB3L1 and CREB3L2 affects Golgi structure

**FIGURE 6:**
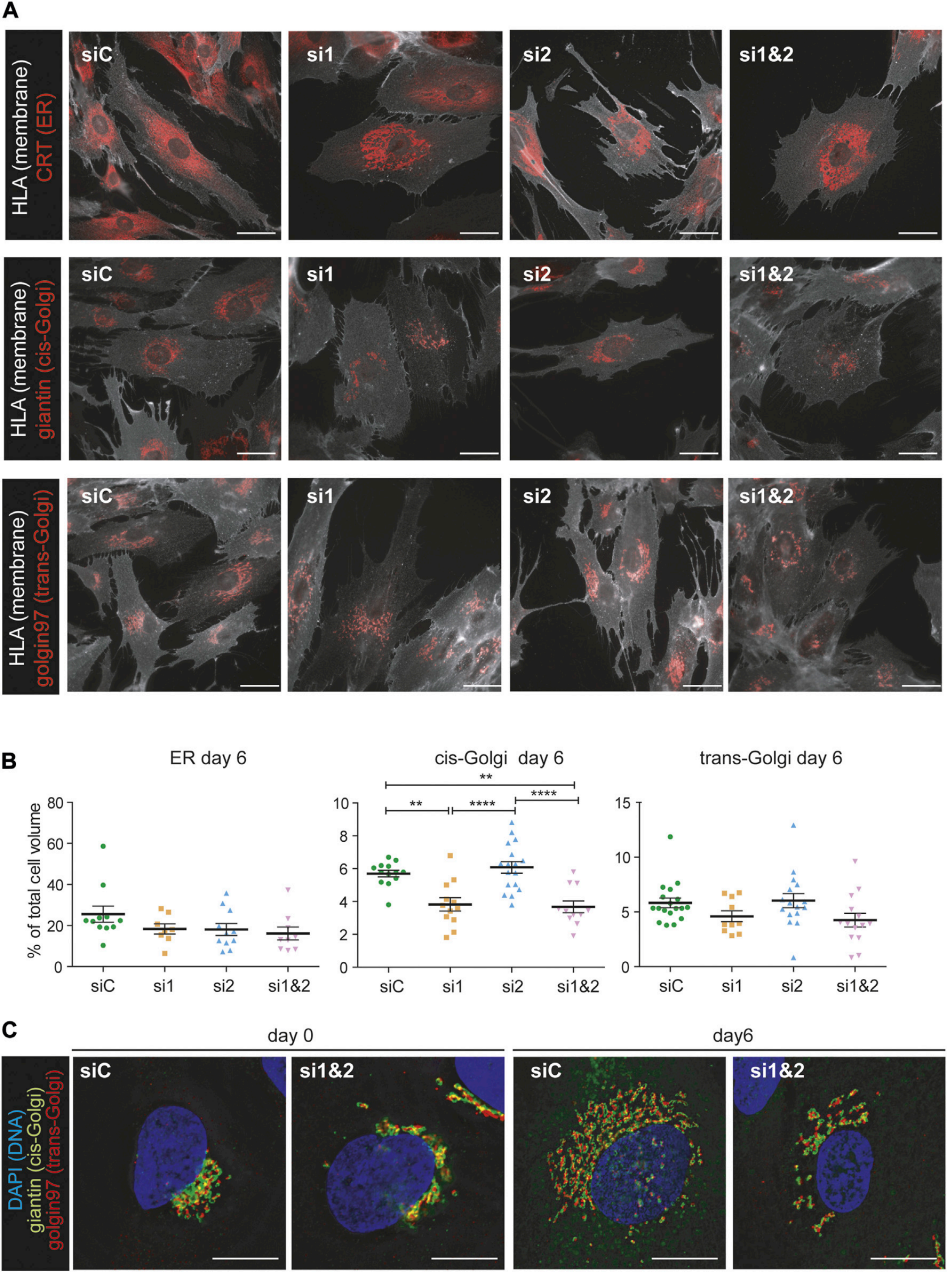
CREB3L1 and CREB3L2 drive the decidualization induced rearrangement of the Golgi compartment. **(A)** T-HESC were treated with scramble duplexes (siC), with duplexes specific for CREB3L1 (si1), CREB3L2 (si2) or both (si1&2). The day after treatment (day0) cells were fixed with PFA or treated with cAMP and progesterone and then collected at day 6 of decidualization. Cells were first decorated with anti-HLA1 antibody to stain the membrane, and then permeabilized and stained with antibodies against different compartment of the early secretory pathway (calreticulin CRT as a marker of the ER, giantin for the cis-Golgi and golgin97 for the trans-Golgi). Representative images of cells at day 6 of differentiation are shown. Images were acquired with a ×60 objective; bar: 20 µm. **(B)** The volume of the cells and of the intracellular compartments was calculated using morphometric analysis of images as the ones shown in **(A)**. The volume of the intracellular compartments was then expressed as a percentage of the total cell volume. The plots show the volume of the ER, cis-Golgi and trans-Golgi of cells at day 6 of decidualization. Each dot in the plot corresponds to a single cell. The plot shows the population distribution and the value of the mean ± SEM for cells of two independent experiments (green: siC, orange: si1, light blue: si2, pink: si1 and 2). *p < 0.05; **p < 0.01, ****p < 0.001, one-way ANOVA with Turkey correction. **(C)** Morphology of the Golgi compartment of cells treated with scrambled duplexes (siC) of KD for both CREB3L1 and 2 (si1&2) at day 0 or day 6 of decidualization. Markers of the cis-Golgi (giantin, green) and of the trans-Golgi (golgin97, red) were used. The DNA is stained in blue (DAPI). Images were acquired with a ×100 objective; bar: 20 µm. Note the fragmentation and the inhibited enlargement of the Golgi compartment under KD of both CREB3L1 and 2. The scattering effect is visible also at day 0.

To better understand the structural changes of the secretory pathway following CREB3L1/2 silencing, we performed morphometric analyses of cells at day 0 and day 6 of decidualization, using calreticulin (CRT) as a marker of the ER, giantin as cis-Golgi marker, and golgin 97 as trans-Golgi marker. Cells were also stained with a membrane marker (HLA 1) to calculate the total cell volume. Silencing of either CREB3L1, 2, or both did not impact on the overall increase in cell volume that characterizes decidualization (Supplementary Figure S2). When expressed as a percentage of the total cell volume, ER size was not affected by the silencing, even if some morphological alterations were evident (Figures 6A upper panels, B). The volume of both the cis-Golgi and the trans-Golgi were instead severely affected by CREB3L1 silencing, both before (Supplementary Figure S2) and after 6 days of decidualization (Figures 6A,B).

Moreover, morphological changes of the Golgi complex were detected, both ad day 0 and at day 6 of decidualization under depletion of one or both TFs (Figure 6C and Supplementary Figure S3). While downregulation of CREB3L1 seems to reduce the Golgi dimension (as indicated by morphometry analyses), CREB3L2 KD seems to cause Golgi fragmentation (Supplementary Figure S3). Combined CREB3L1 and CREB3L2 ablation causes a dramatic decrease of the organelle size, as well as fragmentation and scattering of its *cisternae,* both in resting (day 0) and fully decidualized (day 6) conditions (Figure 6C).

### Impaired secretion upon ablation of CREB3L1 and CREB3L2

**FIGURE 7:**
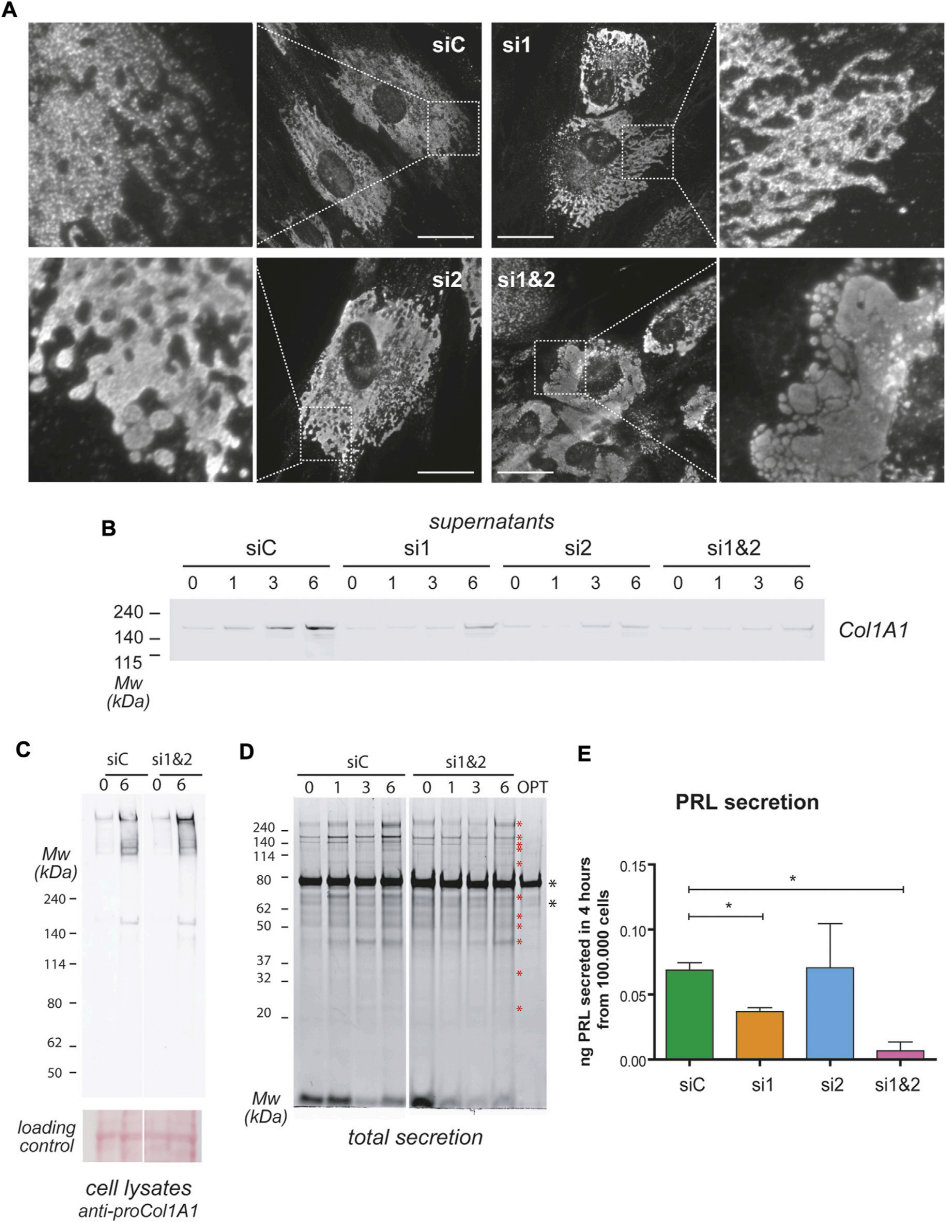
CREB3L1 and 2 KD affects general protein secretion. T-HESC were treated with scramble duplexes (siC), with duplexes specific for CREB3L1 (si1), CREB3L2 (si2) or both (si1&2). The day after treatment (day0) cells were treated with cAMP and progesterone and analyzed at different days of the decidualization process as indicated. **(A)** Images of cells at day 6 of differentiation. Cells were fixed with PFA, permeabilized and stained with anti Pro-Collagen1A1 antibody. In control cells, procollagen is present in the ER. The KD of CREB3L1 and/or 2 dramatically increases the amount of intracellular Pro-Collagen1A1, with swelling of the ER, culminating in Russell Bodies-like structures in conditions of double KD. Images were acquired with a ×60 objective; bar: 20 µm. **(B)** Cells at the indicated days of differentiation were cultured for 6 h in OPTIMEM. The SN corresponding to 10.000 cells were loaded on SDS-PAGE under reducing conditions and, after transferring to nitrocellulose, stained with antibodies against Collagen1A1. The KD of CREB3L1 and/or 2 affects Collagen1A1 secretion. **(C)** The lysates of control cells or of cells subjected to double KD were loaded under non-reducing conditions on SDS-PAGE (3–8% precasted gradient gel), and nitrocellulose was decorated with anti Pro-Collagen1A1. Note the intracellular accumulation of Pro-Collagen1A1 in high molecular weight complexes in conditions of double KD. The ponceau staining of the nitrocellulose is shown as a loading control. **(D)** The absence of CREB3L1 and two affects total protein secretion. The total SN of 10.000 cells treated as indicated were loaded on a precast 4–12% polyacrylamide gel under reducing conditions (SN volumes were normalized with OPTIMEM). Optimem alone (OPT) was loaded as a control. Gels were stained with SyproRuby to stain total secreted proteins. During the decidualization process, protein secretion increases [as previously described (Anelli et al., 2021)]. Asterisks indicate bands that are less present in the double KD SNs. **(E)** PRL secretion was measured with ELISA test in the 4 h SN of day 6 cells treated as indicated. Data as expressed as ng of PRL secreted in 4 h by 100.000 cells. Average of three independent experiments ± SEM. *p < 0.05.

EnSCs express various types of collagen, and their expression changes during decidualization. Under basal conditions, EnSCs express collagen type VI in large amounts, but in the course of decidualization, collagen VI is downregulated, while collagen type I, IV and VIII are upregulated (Figure 2D). CREB3L1 and 2 have been previously identified as necessary for collagen type I and II secretion in osteoblasts and chondrocytes (Murakami et al., 2009; Saito et al., 2009; Chen et al., 2014). We therefore analysed trafficking of collagen type I (intracellular content and localization, and secretion) in decidualizing T-HESC upon silencing of either CREB3L1, 2, or both (Figure 7). Ablation of CREB3L1/2 dramatically affected the fate of collagen type I in decidualizing T-HESC (Figure 7A), causing the formation of long stacks or enlarged lacunae in the endoplasmic reticulum. The trafficking defect is particular evident in the double KD, with the formation of abnormal structures, filled with ProCol1A1 and positive for calreticulin (not shown), that are reminiscent of aggregated Ig-containing Russell Bodies (Valetti et al., 1991). In accordance, ablation of CREB3L1/2 led to high MW collagen complexes in cell lysates (Figure 7C), which is indicative of the intracellular accumulation of collagen aggregates. Consequently, collagen type I secretion was almost completely abrogated (Figure 7B), in particular upon double silencing. Moreover, ablation of CREB3L1/2 caused an overall reduction in the secreted material of decidualizing T-HESC (Figure 7D). For instance, PRL, which normally is abundantly secreted upon decidualization, was no longer detectable in the supernatants of double CREB3L1 and 2 KD cells (Figure 7E). This suggests a more general effect on total protein trafficking and secretion.

## Discussion

We previously described how decidualization of EnSC goes hand in hand with morphological changes of the entire secretory pathway, which include rearrangements not only of the ER but also of the Golgi compartment (Anelli et al., 2021). Here, by transcriptomics analysis of *in vitro* T-HESC differentiation, we employed an “omic” approach to dissect the gene expression changes occurring during decidualization. Our current study confirms in further detail that decidualization entails the upregulation of genes associated with several components of the secretory pathway. Moreover this analysis reveals how decidualization stimuli induce a readjustment of the secretory cargoes, with not only an increase in the amount of cargoes produced, but also a change in the types of collagens and laminins synthesized (Figure 2D). This is in line with previous studies which suggested that a change in the composition of the extracellular matrix is needed to accomodate the incoming embryo (Iwahashi et al., 1996; Shi et al., 2020). Our results furthermore highlight that, even if decidualization intails a dramatic increase in the secretory activity, proliferating EnSC are already active secretors, specifically releasing components of the extracellular matrix. Moreover, activation of cAMP-dependent pathways and the upregulation of genes of the early secretory pathway can be detected early during decidualization, even before the upregulation of secretory cargoes (Figures 2C,D). Evidently, this is due to the activation of a differentiation program and is not a cargo-induced effect: decidualizing EnSCs first adapt and reshape their secretory machinery and later up-regulate their cargoes.

**FIGURE 8:**
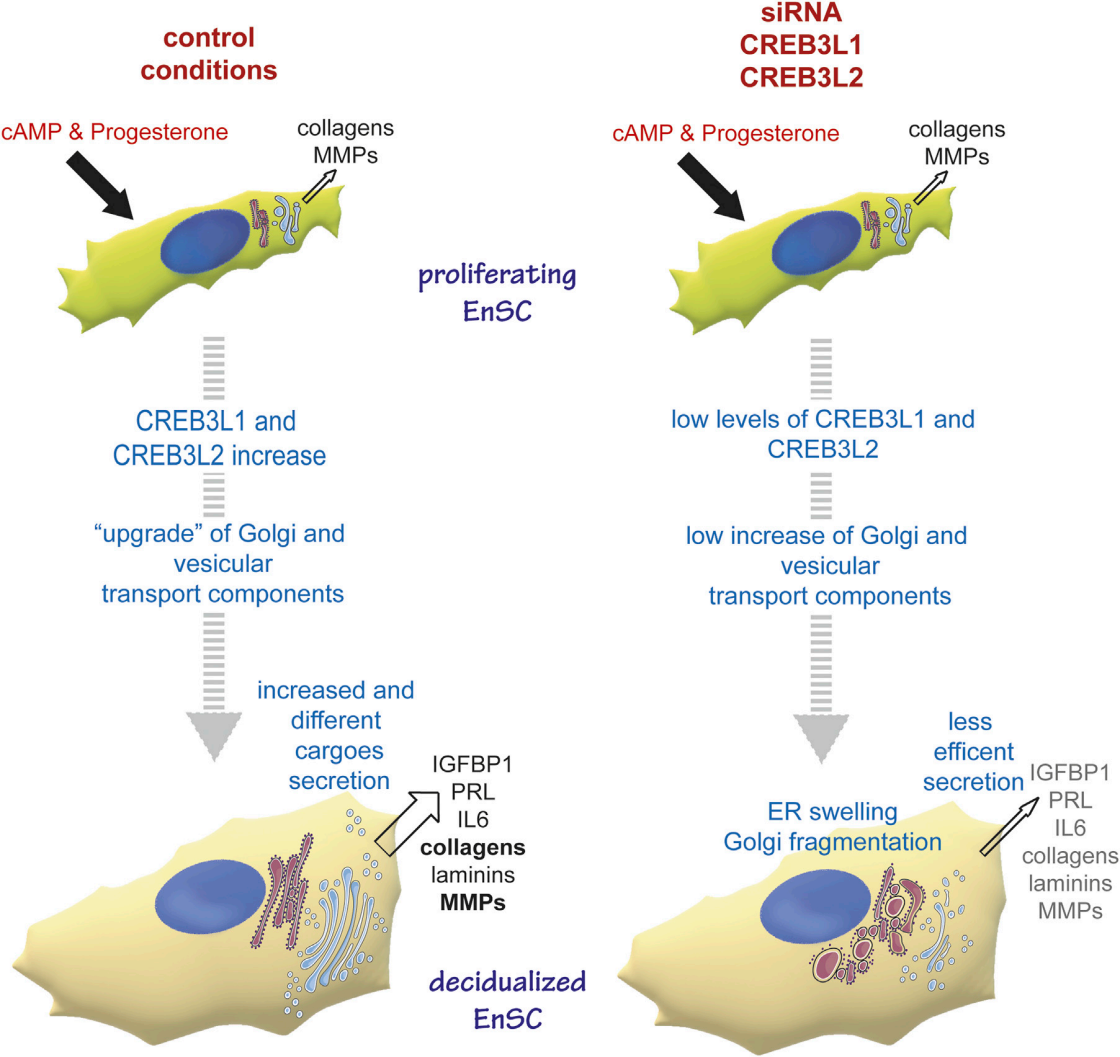
CREB3L1 and 2 mediate the reshaping of the early secretory pathway during decidualization. Under proliferating conditions, EnSC produce and secrete some collagens and MMPs. The decidualising stimulus induces upregulation and activation of CREB3L1 and CREB3L2, which have consequences on cell morphology, with a reshaping of the early secretory pathway. Thus Golgi and ER to Golgi vesicular transport components are up-regulated, which ensures more efficient secretion of new and different cargoes. In conditions of CREB3L1 and/or 2 KD, the cells are not able to adjust their secretory machinery to the new cargo load and production. Hence the ER swells, Golgi morphology is profoundly affected (with no enlargement and cisternae fragmentation) and secretion in general is lowered.

Clustering analysis showed that genes of the early secretory compartment specifically involved in ER to Golgi trafficking and Golgi structure are upregulated with a similar pattern, which suggests shared transcriptional regulation(s). Promoter analyses revealed a potential contribution of ATF6, CREB3L1 and CREB3L2. Having shown previously that UPR branches are not overtly activated during decidualization (Anelli et al., 2021), we focused our attention on CREB3L1 and 2, two transcription factors that are known to regulate collagen secretion (Saito et al., 2009; Chen et al., 2014). These factors are also required in some cell types that do not produce collagens. Indeed, CREB3L1 is upregulated in thyrocytes upon TSH stimulation to sustain Golgi enlargement (Garcia et al., 2017). CREB3L2 instead is needed for hepatic stellate cells differentiation to myoblasts-like cells, a process requiring ER and Golgi enlargement (Tomoishi et al., 2017), as well as for maturation of pituitary cells into hormone producing factories (Khetchoumian et a., 2019). CREB3L2 has been suggested as a master TF for the control of secretory capacity of cells (Khetchoumian et a., 2019). CREB3L1 and CREB3L2 Drosophila Melanogaster homologue, CREB3A, controls the transcriptional activation of many genes encoding the core secretory pathway (Johnson et al., 2020). We show here that, in EnSCs, both CREB3L1 and CREB3L2 sustain the expression of SEC24A and D, GOLGB1 and KDELR2 and 3 even under basal conditions (Figure 5). Moreover, they are needed for organelle remodelling upon progesterone stimulation. Upon CREB3L1 ablation, the Golgi complex does no longer enlarge, while the ablation of CREB3L2 results in Golgi scattering. Thus, CREB3L1 and CREB3L2 jointly regulate both Golgi volume and structure (Figure 6), and, consequently, are key for efficient protein secretion (Figure 7 and Figure 8). The changes in ER morphology upon CREB3L1 and/or 2 KD are also striking (Figure 6 and Figure 8). Whether the anomalous swelling of the ER is a direct consequence of the ablation of the two TFs, or whether it is due to impaired collagen trafficking and accumulation is not clear and cannot be easily addressed, given the high diversity of collagens produced by EnSCs. No canonical UPR activation is observed under condition of CREB3L1 and/or 2 ablation at day 0 (not shown), even if their KD still have consequences both at a transcriptional and at a morphological level.

Up to now, defects of CREB3L1 or CREB3L2 have been linked to skeletal disorders. CREB3L1 mutations are associated with Osteogenesis Imperfecta (Symoens et al., 2013; Keller et al., 2018; Lindahl et al., 2018), while CREB3L2^KO^ mice, instead, display a severe chondrodysplasia (Saito et al., 2009). Differently from osteoblasts or chondrocytes, our results indicate that decidualizing cells rely on both CREB3L1 and 2. We could speculate that this is due to dEnSC producing and secreting different cargoes, including various collagen types. We cannot exclude that in EnSC CREB3L1 and 2 could also heterodimerize, as previously described (Khan and Margulies, 2019).

The mechanism by which CREB3L1 and 2 are activated has been well characterized. Both must exit the ER to be cleaved by S1P and S2P at the Golgi: this process liberates the cytosolic portions that translocate to the nucleus to become transcriptionally active, analogous to the activation mechanism of ATF6 and SREBP proteins (Kondo et al., 2007; Murakami et al., 2009). What drives the exit of CREB3L1 and 2 from the ER is, however, not completely understood yet. In fibroblasts and HeLa cells, CREB3L1 and 2 are continuously rapidly degraded, such that their up-regulation allows a fraction of the CREB3L1 and 2 pool to escape from the degradation machinery and, hence, exit from the ER to the Golgi, leading to their activation (Kondo et al., 2012). Whether such a scenario holds true for the decidualization process awaits further analysis. While both CREB3L1 and 2 are transcriptionally induced in stimulated EnSC, only CREB3L1 increases also at the protein level, suggesting that different processing mechanisms may apply to either of the two TF. Androgen receptor activates CREB3L2, increasing protein trafficking in prostate cancers (Hu et al., 2021), but the biochemical details remain to be elucidated. It is tempting to speculate that similar mechanisms are shared by progesterone receptor, which will be a topic for further studies.

Altogether, our data shed new light on the molecular mechanisms underlying the reshaping of the secretory pathway during decidualization. With their unique cargo heterogeneity, EnSCs behave differently from other known cell types differentiating towards *protein factories*. Thus, they prove to be an invaluable model to study adaptation of the secretory pathway during differentiation towards a highly secretory phenotype.

## Materials and methods

### Reagents and antibodies

Chemicals and reagents were purchased from Sigma-Aldrich (St Louis, MO), unless otherwise specified. Custom oligonucleotides were purchased from Metabion International AG (Planegg, Germany). The following primary antibodies were used: mouse monoclonal anti-ERp44 36C9, that has been previously described (Anelli et al., 2003; Anelli et al., 2007); mouse monoclonal anti-HLA1 W6/32 (ATCC); mouse monoclonal anti-actin, rabbit anti-CRT, goat anti-Vimentin, rabbit anti-ERGIC-53 and Phallodin-FITC (Sigma, St Louis, MO); mouse monoclonal anti-proCOL1A1 (Developmental Studies Hybridoma Bank [DSHB]); rabbit polyclonal anti-Col1A1 and mouse monoclonal anti-golgin 97 (CDF4) (Invitrogen Molecular Probes [Eugene, OR, United States]); mouse monoclonal anti-GM130 (BD Biosciences, Franklin Lakes, NJ); rabbit polyclonal anti-giantin (19243) (Biolegend, San Diego, CA); Alexa Fluor conjugated secondary antibodies (488, 647 and 700) were from Invitrogen Molecular Probes (Eugene, OR). CREB3L1 and CREB3L2 were detected respectively using the following antibodies: anti-OASIS, clone 10H1, MABE1151 (EMD corp., United States); anti-BBF2H7, clone 28G9, MABE1018 (EMD corp., United States).

### Cell lines

Experiments were performed on the human immortalized EnSC cell line T-HESC, obtained from ATCC (ATCC CRL-4003). Cells were cultured, according to manufacturer’s instructions, in a 1:1 mixture of Dulbecco’s modified Eagle’s medium and Ham’s F-12 medium without Phenol Red, containing 3.1 g/L glucose and 1 mM sodium pyruvate, and supplemented with 1.5 g/L sodium bicarbonate, 1% ITS + Premix (Corning), 500 ng/ml puromycin, and 10% charcoal/dextran treated fetal bovine serum. Medium was supplemented every 3–4 days with 50 ug/ml ascorbic acid. Decidualisation was induced by adding in the medium cAMP (8-bromoadenosine 3′, 5′ cyclic monophosphate) 0.5 mM and MPA (medroxyprogesterone) 1uM as previously described (Anelli et al., 2021). HeLa cells were maintained in High Glucose DMEM supplemented with 10% fetal bovine serum and with penicillin/streptomycin (100 U/ml and 100 ug/ml respectively).

### Bulk RNA-seq time course analysis and clustering

Cell samples were collected in duplicate before the stimulus (0 h) and at time 6, 18 h, d1, d1.5, d2, d3, d6 after the start of the decidualization protocol. RNA was harvested using TRIzol (Invitrogen) according to the manufacturer’s instructions. Library preparation was created from 500 ng of total RNA per sample and processed using the TruSeq Stranded mRNA kit, according to the manufacturer’s protocol. The sequencing step was carried on an Illumina HiSeq 3,000 platform. Sequencing data were analyzed using snakePipes with standard parameters (Bhardwaj et al., 2019). The pipeline employs STAR aligner to map the reads to the human genome (genome build hg38). FeatureCounts was utilized to count the number of reads per annotated gene using gencode gtf file (release 27). Raw sequencing data and raw counts are available on the Gene Expression Omnibus repository, accession number: GSE200200. Differential expression analysis (DEA), over-representation analysis (ORA), time series clustering and data visualization were performed using the R programming language (v. 4.1.0). Count matrix was prefiltered before DEA by removing all genes showing a total number of counts, across al the samples, less than 30. DEA design consists of a pairwise comparison of each timepoint replicates *versus* untreated ones using the DESEQ2 package (v. 1.32.0) (Love et al., 2014). DEGs were called based on adjusted *p*-value < 0.01 and absolute log_2_ fold change >0.5. ORA of DEA outputs was carried out on the enrichR package (v. 3.0). Enrichment for Gene Ontology (GO) terms was performed based on the database GO molecular function, cellular component, biological pathways, 2021 release. Candidate genes for time series clustering of ER and Golgi apparatus compartments were selected for both of the following criteria: being called differentially expressed at any timepoints ranging from 6 to 72 h and being annotated to GO cellular component terms GO:0005783 Endoplasmic Reticulum and/or GO:0005794 Golgi Apparatus, 2018 release. Annotations were downloaded from AmiGO2, setting “*Homo Sapiens*” and “experimental evidence” as filtering criteria parameters for “organism” and “evidence”, respectively. The input matrix for clustering was retrieved by subsetting the time-course expression of the genes of interest from the original expression matrix transformed using DESEQ2 variance stabilizing transformation. Then, expression value information for each time point was obtained by averaging replicates of the same time point. Last, we performed z-normalization to optimize the performance of the distance metric utilized in the clustering. Time-series clustering was performed using the dtwclust package (v. 5.5.10) (Sardà-Espinosa, 2019). The shape-based distance (SBD) proposed by Paparrizos and colleagues (Paparrizos and Gravano, 2015) and partition around medoids (PAM) were chosen as the distance metric and clustering algorithm, respectively. SBD consists of a normalized version of the cross-correlation measure, which allows for determining the similarity of the shape between time signals, even if they are not properly aligned. PAM requires to set a k number of clusters *a priori*. The optimal number of k clusters was determined with a heuristic approach based on the visual inspection of the clusters that maximized the average Silhouette index. The presented clustering output was obtained by choosing k = 5 and the seed set to 23. To polish the clusters from genes showing dramatic out of phase temporal expression, we removed from the clustering output all genes showing, at any time point, an absolute z-score expression value higher or lower than two standard deviations from the mean z-score expression for the considered timepoint. Enrichment for BP GO terms for each cluster was carried out on topGO package (v.2.44.0) (Alexa and Rahnenfuhrer, 2022). ORA was performed by adjusting the gene Universe to the genes contained in the polished version of the clustering output. The weighted algorithm proposed in Alexa et al., 2006 was used to reduce the impact of local dependencies in the GO graph. Enrichment for shared transcription factor binding motif (TFBM) for the genes in each cluster was performed using RcisTarget (v. 1.12.1) (Aibar et al., 2017). The analysis was performed by evaluating the shared motifs found at ± 10 kb from the transcription start site of the genes in the input list. Data visualization was performed using the packages ggplot2 (v. 3.3.6) (Wickham et al., 2016) and pheatmap (v. 1.0.12) (Raivo Kolde, 2019).

### siRNA and transient transfection

Silencing was performed with Lipofectamine RNAiMax reagent (Thermofisher) according to manufacturer’s instruction. CREB3L1 was silenced with a custom siRNA oligonucleotide [5′CCA​CCA​AGU​ACC​UGA​GUG​A (dT)(dT)-3′, sense], while for CREB3L2 a previously described siRNA oligonucleotide was used [5′GAG​UCU​UGU​UCA​ACU​GAG​A (dT)(dT)-3′, sense] (Tomoishi et al., 2017). Non-targeting duplexes used as control (SiC) were purchased from Darmachon (Lafayette, Colorado, United States) (catalog number D-001810–01). Silencing of the genes of interest was confirmed by RealTime qPCR, as described below.

HeLa cells were transfected with JetPEI transfection reagent (Euroclone) according to manufacturer’s instructions.

### Cloning of cytosolic CREB3L1 and CREB3L2

Cytosolic domains of CREB3L1 and CREB3L2 were cloned from RNA of T-HESC cell line, retrotranscribed with RT Polymerase (Promega) and oligo-dT primers (Promega) according to manufacturer’s instructions. To ensure high expression of the transcripts and avoid cross-contamination, RNAs at day 3 of decidualization with silencing of CREB3L2 and CREB3L1 (confirmed by RealTime quantitative PCR) were used to amplify CREB3L1 and CREB3L2 respectively. PCR was performed with Pfu DNA Polymerase (Promega), with the following conditions: 95°C 2 min; 95°C 30 s, 52°C (CREB3L1)/56°C (CREB3L2) 30 s, 72°C 3 min × 35 cycles; 72°C, 5 min (CREB3L1 fw: CTA​GCT​AGC​TAG​GGC​TGC​GAT​GGA​CGC​CGT​C; rev: CCG​GAT​ATC​TTA​TTA​TGG​TCC​CAG​TCT​GGG​TG; CREB3L2 fw: CTA​GCT​AGC​TAG​GGC​CCG​CAC​CGC​CAT​G; rev: CCG​GAT​ATC​TTA​TTA​GCA​GGT​GCC​AGT​CTG​CGT​G). Vector pcDNA3.1 (+) and PCR products were digested with NheI-HF (Biolabs) and EcoRV (Biolabs), run in 1% agarose gel and purified (ZymoClean Gel DNA Recovery Kit, Zymo Research). Ligation was performed with T4 DNA ligase (Promega), with a 3:1 insert/plasmid ratio. Plasmid identity was then confirmed by site-specific enzymatic digestion with NotI (Biolabs) and BstxI (Promega) and by sequencing (Metabion, Planegg/Steinkirchen Germany).

### Western blot analysis

Western Blot assay was performed as previously described (Anelli et al., 2021). Briefly, cells were detached with Trypsin/0.5% EDTA (ThermoFisher) and counted with Neubauer chamber, washed once in ice-cold PBS (Gibco) and once in ice-cold PBS containing 10 mM N-ethylmaleimide (NEM) to block rearrangement of disulfide bonds; lysis was performed in 150 mM NaCl, 1% NP-40, 2% SDS, 50 mM Tris HCl pH 7.4 containing 10 mM NEM, cOmplete EDTA-free protease inhibitor cocktail (Roche) and phosphatase inhibitors, using 10uL of lysis buffer for 100.000 cells. Samples were processed with Benzonase nuclease according to manufacturer’s instruction. Samples were then loaded in reducing conditions (50 mM DTT) on SDS-PAGE (4–12% or 3–8% precast gel, Invitrogen), normalizing by protein quantity (40 ug/lane), unless differently specified. Western blot in Figure 6C was instead performed in non-reducing conditions. After transfer on nitrocellulose (0.2 um, Ahmersham), membranes were saturated in 5% milk in PBS 0.1% Tween and then incubated with specific antibodies as indicated (primary antibodies 1:500 in PBS 0.1% tween, 2% milk; secondary antibodies Alexa Fluor conjugated [647 or 546] 1:1,000 in PBS 0.1% Tween; HRP-conjugated secondary antibodies 1:5,000 in PBS-0.1% tween). Signals were detected by fluorescence, with FujiFilm FLA 9000 (FujiFilm Life Science, Tokyo, Japan), or by chemoluminescence, with Uvitec Alliance Mini HD9 (Cambridge, United Kingdom). Densitometric analysis of the images was performed with ImageJ. Signals were normalized on protein loading (ponceau staining).

### Secretion assay and SYPRO Ruby staining

For secretion assays, at the end of the siRNA and decidualization period, the cells were washed three times in PBS and incubated for 6 h in serum free medium (OPTIMEM) (Gibco) containing 50 ug/ml Ascorbic Acid (final concentration). The 6-h culture supernatants were collected and centrifuged once at 1000 g to get rid of detached dead cells; then they were added with 10 mM NEM and cOmplete EDTA-free protease inhibitor cocktail (Roche). Cells were detached, counted and lysed as described above. A volume of the supernatants corresponding to equal cell numbers was loaded on SDS-PAGE (4–12% precast gel, Invitrogen). The gels were then either used for Western Blot analysis (as described above), or stained with SYPRO Ruby Protein Gel Stain (Merck) according to the manufacturers’ protocols. For SYPRO Ruby staining, fluorescence signals images were acquired with FujiFilm FLA 9000 (FujiFilm Life Science, Tokyo, Japan) and processed with ImageJ.

### Prolactin measurements

For PRL measurements, cells (control or subjected to siRNA as indicated) at day 6 of decidualization were incubated for 4 h in OPTIMEM. Cell culture supernatants were collected and cells were detached and counted. PRL concentration in the spent medium was measured using a two-steps Sandwich ELISA. Briefly, plate wells were coated with capture antibody (anti-human PRL Goat IgG RD System, 800 ng/ml 100 µL/well) O/N at 4°C. After three washes in 300 µL of wash buffer (WaBu, PBS 0.05% Tween 20), saturation was performed in PBS 1% BSA (300 µL/well). Blocking was discarded and samples (100 µL of 6 h SN in OPTIMEM) were added, O/N at 4°C. Purified human PRL (RD System) diluted in PBS was used as standard curve, in a range from 0.01 ng/100 μL to 5 ng/100 µL. OPTIMEM alone (100 µL/well) was used as a negative control. After three washing in 300 µL of WaBu, wells were treated with a biotinylated detection antibody (Goat anti-human PRL IgG RD System, 400 ng/ml, 100 µL/well, 2 h RT). Wells were washed 3 times with 300 µL of WaBu, and then treated with HRP conjugated Streptavdin (RD System, DY998) (1:200, 100µL/well, 1 h RT). After three washings with 300 µL of WaBu, the HRP substrate (SigmaFast OPD, Sigma) was added (100 µL/well, 20 min RT) and the optical density was measured at 450 nm. The amount of secreted PRL was expressed as a ratio on the cell number.

### Immunofluorescence and morphometric analysis

Cells were plated at a suitable confluence directly on glass slides. After treatments, cells were fixed in 4% PFA for 10 min at RT and washed in PBS. For conventional immunofluorescence, cells were permeabilized with 0.1% Triton X-100 in PBS, incubated with blocking solution (PBS-5% FCS) for 30 min at RT, and then incubated with primary and secondary antibodies (Alexa Fluor conjugated 488 or 647) or with Phalloidin-FITC (Sigma), 1:500 in PBS-5% FCS, for 30 min at RT. For staining with anti-golgin 97 cells were permeabilized in 0.1% saponin, and the detergent was kept for all the subsequent steps of staining. Nuclei were stained with DAPI (Sigma) (1:5,000), and coverslips were mounted using Mowiol mounting medium. For morphometry, cells were first incubated with blocking solution, anti-HLA antibody (W6-32), and fluorescent secondary antibody before permeabilization; immunofluorescence for the intracellular proteins was then performed as described above. Images were acquired with DeltaVision GE healthcare DeltaVision Ultra microscope (ALEMBIC, Milan, Italy), equipped with oil lenses. A ×60 magnification (Olympus ×60/1.42, Plan Apo N, UIS2, 1-U2B933) was used for morphometry, while a ×100 magnification (Olympus ×100/1.40, UPLS Apo, UIS2, 1-U2B836) was used for organelle morphology analysis. For morphometry analyses, images were deconvolved with Huygens Professional version 19.04 (Scientific Volume Imaging, Hilversum, The Netherlands, http://svi.nl), using the CMLE algorithm, with SNR:10 and 40 iterations. Analyses of Golgi, ER and total cell volume were performed with the advanced Object Analyzer plugin of the Huygens Professional software (garbage 1, seed value 10%, and threshold 4%). With this strategy, cell volume, ER or Golgi volume and organelle/cell volume ratio were determined for each cell.

### PCR and quantitative real-time PCR

Semi-quantitative real-time PCR (qRT-PCR) amplification was performed (as described in Sanchez et al., 2016) with the SYBR PCR Master Mix kit (Applied Biosciences, Waltham, MA) using the Biorad Real Time PCR machine (CFX96 Real Time System). Briefly, 1uL of primers (final concentration 2,5 uM), 4 ng of template DNA in nuclease free water and 5uL of Master Mix were added to each reaction. All the samples were loaded in technical triplicate, and at least in biological duplicate, Cycling was performed with the following conditions: preincubation 10 min 95°, amplification 40 cycles 15″ 95° + 60″ 60°. Melting curves were evaluated for each gene. Relative quantification of gene expression was performed for each condition: GAPDH was chosen as a reference gene, since it is constant during all the days of decidualization. Mock-transfected, undifferentiated cells (D0 siC) were considered as control condition, and the expression of each gene was expressed as an increase (or decrease) relative to the value measured at D0 siC (ΔΔCT). The results were expressed as fold gene expression values (2−ΔΔCT), averaged and plotted in the graphs.

qRT-PCR primers used for KDELRs were obtained from Qiagen (Hilden, Germany): Hs_KDELR1_1_SG QuantiTect Primer Assay (QT00090811) for KDELR1, Hs_KDELR2_1_SG QuantiTect Primer Assay (QT00092715) for KDELR2, Hs_KDELR3_1_SG QuantiTect Primer Assay (QT00097790) for KDELR3. Other primers were purchased from Metabion (Planegg, Germany) and are listed in Table 1.

**TABLE 1:** Primer list.

Gene	Primer forward	Primer reverse
GAPDH	TGA​AGG​TCG​GAG​TCA​ACG​GAT​TT	CAT​GTA​AAC​CAT​GTA​GTT​GAG​GT
CREB3L1	ACA​ATG​CGC​ACT​TTC​CTG​AG	GAG​GGC​TCT​TCT​CAT​CCA​GC
CREB3L2	AGA​ATA​CAT​GGA​CAG​CCT​GGA​G	TCT​AGA​ACC​TCT​ACC​TTC​TTC​CGA
ERP44	CAG​CAC​TCT​GAC​ATA​GCC​CA	CTG​ATC​GCT​GAC​CCC​TGT​AT
LMAN1	TCA​GGA​GGA​ATT​TGA​GCA​CTT​T	GCT​CTC​GAT​CTC​CTA​CAC​TCT​CA
GOLGA2	CAGCCGCCTGCAGTATTC	TGA​GGG​CGT​CTC​TCT​CTT​TG
GOLGB1	AGC​ATC​TCA​GAC​TTC​TTT​CCC​A	GAG​CAA​GGC​TCC​CTT​TTC​AT
TGOLN2	GAG​CAG​CCA​CTT​CTT​TGC​AT	TTC​CTT​CCA​GGA​CAA​AAG​CA
SEC24A	ACA​CTG​GAG​GCT​GAG​GTG​G	ACA​CTG​GAG​GCT​GAG​GTG​G
SEC24D	ACC​CCA​TCA​GTT​TGG​TCA​GA	CCA​CAT​TGT​TGA​CAG​GTG​GA
PRL	AGC​CAG​GTT​CAT​CCT​GAA​A	AGC​AGA​AAG​GCG​AGA​CTC​TT

### Statistical analysis

Statistical analysis for wet biology experiments was performed using a two tails unpaired student’s *t* Test with Welch’s correction or, in case of multiple comparisons, with one-way Anova with Turkey correction. *p* values ≤ 0.5 were considered significant. Statistics were obtained using Graph Pad Prism (6.0 version).

## Data Availability

The datasets presented in this study can be found in online repositories. The names of the repository/repositories and accession number(s) can be found below: GEO accession: GSE200200.

## References

[B1] Aibar S. Gonzalez-Blas C. B. Moerman T. Huynh-Thu V. A. Imrichova H. Hulselmans G. (2017). Scenic: Single-cell regulatory network inference and clustering. Nat. Methods 14, 1083–1086. 10.1038/nmeth.4463 28991892 PMC5937676

[B2] Alexa A. Rahnenfuhrer J. Lengauer T. (2006). Improved scoring of functional groups from gene expression data by decorrelating GO graph structure. Bioinformatics 22, 1600–1607. 10.1093/bioinformatics/btl140 16606683

[B47] Alexa A Rahnenfuhrer J (2022). topGO: Enrichment Analysis for Gene Ontology. R package version 2.48.0.

[B3] Anelli T. Dalla Torre M. Borini E. Mangini E. Ulisse A. Semino C. (2021). Profound architectural and functional readjustments of the secretory pathway in decidualization of endometrial stromal cells. Traffic 23, 4–20. 10.1111/tra.12822 34651407

[B4] Anelli T. Panina-Bordignon P. (2019). How to avoid a No-deal ER exit. Cells 8, E1051. 10.3390/cells8091051 31500301 PMC6769657

[B5] Anelli T. Sitia R. (2008). Protein quality control in the early secretory pathway. Embo J. 27, 315–327. 10.1038/sj.emboj.7601974 18216874 PMC2234347

[B6] Asada R. Kanemoto S. Kondo S. Saito A. Imaizumi K. (2011). The signalling from endoplasmic reticulum-resident bZIP transcription factors involved in diverse cellular physiology. J. Biochem. 149, 507–518. 10.1093/jb/mvr041 21454302

[B7] Bhardwaj V. Heyne S. Sikora K. Rabbani L. Rauer M. Kilpert F. (2019). snakePipes: facilitating flexible, scalable and integrative epigenomic analysis. Bioinformatics 35, 4757–4759. 10.1093/bioinformatics/btz436 31134269 PMC6853707

[B8] Braakman I. Bulleid N. J. (2011). Protein folding and modification in the mammalian endoplasmic reticulum. Annu. Rev. Biochem. 80, 71–99. 10.1146/annurev-biochem-062209-093836 21495850

[B9] Carbone K. Pinto N. M. Abrahamsohn P. A. Zorn T. M. (2006). Arrangement and fine structure of collagen fibrils in the decidualized mouse endometrium. Microsc. Res. Tech. 69, 36–45. 10.1002/jemt.20265 16416410

[B10] Chan C. P. Kok K. H. Jin D. Y. (2011). CREB3 subfamily transcription factors are not created equal: Recent insights from global analyses and animal models. Cell Biosci. 1, 6. 10.1186/2045-3701-1-6 21711675 PMC3116243

[B11] Chen Q. Lee C. E. Denard B. Ye J. (2014). Sustained induction of collagen synthesis by TGF-beta requires regulated intramembrane proteolysis of CREB3L1. PloS one 9, e108528. 10.1371/journal.pone.0108528 25310401 PMC4195586

[B12] Christianson J. C. Carvalho P. (2022). Order through destruction: How ER-associated protein degradation contributes to organelle homeostasis. EMBO J. 41, e109845. 10.15252/embj.2021109845 35170763 PMC8922271

[B13] Christis C. Fullaondo A. Schildknegt D. Mkrtchian S. Heck A. J. Braakman I. (2010). Regulated increase in folding capacity prevents unfolded protein stress in the ER. J. Cell Sci. 123, 787–794. 10.1242/jcs.041111 20144991 PMC2823579

[B14] Federovitch C. M. Ron D. Hampton R. Y. (2005). The dynamic ER: Experimental approaches and current questions. Curr. Opin. Cell Biol. 17, 409–414. 10.1016/j.ceb.2005.06.010 15975777

[B15] Fox R. M. Hanlon C. D. Andrew D. J. (2010). The CrebA/Creb3-like transcription factors are major and direct regulators of secretory capacity. J. Cell Biol. 191, 479–492. 10.1083/jcb.201004062 21041443 PMC3003312

[B16] Garcia I. A. Torres Demichelis V. Viale D. L. Di Giusto P. Ezhova Y. Polishchuk R. S. (2017). CREB3L1-mediated functional and structural adaptation of the secretory pathway in hormone-stimulated thyroid cells. J. Cell Sci. 130, 4155–4167. 10.1242/jcs.211102 29093023 PMC6518157

[B17] Gellersen B. Brosens J. J. (2014). Cyclic decidualization of the human endometrium in reproductive health and failure. Endocr. Rev. 35, 851–905. 10.1210/er.2014-1045 25141152

[B18] Harding H. P. Zeng H. Zhang Y. Jungries R. Chung P. Plesken H. (2001). Diabetes mellitus and exocrine pancreatic dysfunction in perk-/- mice reveals a role for translational control in secretory cell survival. Mol. Cell 7, 1153–1163. 10.1016/s1097-2765(01)00264-7 11430819

[B19] Hu L. Chen X. Narwade N. Lim M. G. L. Chen Z. Tennakoon C. (2021). Single-cell analysis reveals androgen receptor regulates the ER-to-Golgi trafficking pathway with CREB3L2 to drive prostate cancer progression. Oncogene 40, 6479–6493. 10.1038/s41388-021-02026-7 34611310

[B20] Ishikawa T. Toyama T. Nakamura Y. Tamada K. Shimizu H. Ninagawa S. (2017). UPR transducer BBF2H7 allows export of type II collagen in a cargo- and developmental stage-specific manner. J. Cell Biol. 216, 1761–1774. 10.1083/jcb.201609100 28500182 PMC5461018

[B21] Iwahashi M. Muragaki Y. Ooshima A. Yamoto M. Nakano R. (1996). Alterations in distribution and composition of the extracellular matrix during decidualization of the human endometrium. J. Reprod. Fertil. 108, 147–155. 10.1530/jrf.0.1080147 8958841

[B22] Iwakoshi N. N. Lee A. H. Vallabhajosyula P. Otipoby K. L. Rajewsky K. Glimcher L. H. (2003). Plasma cell differentiation and the unfolded protein response intersect at the transcription factor XBP-1. Nat. Immunol. 4, 321–329. 10.1038/ni907 12612580

[B23] Johnson D. M. Wells M. B. Fox R. Lee J. S. Loganathan R. Levings D. (2020). CrebA increases secretory capacity through direct transcriptional regulation of the secretory machinery, a subset of secretory cargo, and other key regulators. Traffic 21 (9), 560–577. 10.1111/tra.12753 32613751 PMC8142552

[B24] Keller R. B. Tran T. T. Pyott S. M. Pepin M. G. Savarirayan R. Mcgillivray G. (2018). Monoallelic and biallelic CREB3L1 variant causes mild and severe osteogenesis imperfecta, respectively. Genet. Med. 20, 411–419. 10.1038/gim.2017.115 28817112 PMC5816725

[B25] Khan H. A. Margulies C. E. (2019). The role of mammalian creb3-like transcription factors in response to nutrients. Front. Genet. 10, 591. 10.3389/fgene.2019.00591 31293620 PMC6598459

[B26] Khetchoumian K. Balsalobre A. Mayran A. Christian H. Chénard V. St-Pierre J. (2019). Pituitary cell translation and secretory capacities are enhanced cell autonomously by the transcription factor Creb3l2. Nat. Commun. 10 (1), 3960. 10.1038/s41467-019-11894-3 31481663 PMC6722061

[B27] Kondo S. Hino S. I. Saito A. Kanemoto S. Kawasaki N. Asada R. (2012). Activation of OASIS family, ER stress transducers, is dependent on its stabilization. Cell Death Differ. 19, 1939–1949. 10.1038/cdd.2012.77 22705851 PMC3504707

[B28] Kondo S. Saito A. Hino S. Murakami T. Ogata M. Kanemoto S. (2007). BBF2H7, a novel transmembrane bZIP transcription factor, is a new type of endoplasmic reticulum stress transducer. Mol. Cell. Biol. 27, 1716–1729. 10.1128/MCB.01552-06 17178827 PMC1820470

[B29] Lindahl K. Astrom E. Dragomir A. Symoens S. Coucke P. Larsson S. (2018). Homozygosity for CREB3L1 premature stop codon in first case of recessive osteogenesis imperfecta associated with OASIS-deficiency to survive infancy. Bone 114, 268–277. 10.1016/j.bone.2018.06.019 29936144

[B30] Love M. I. Huber W. Anders S. (2014). Moderated estimation of fold change and dispersion for RNA-seq data with DESeq2. Genome Biol. 15, 550. 10.1186/s13059-014-0550-8 25516281 PMC4302049

[B31] Murakami T. Saito A. Hino S. Kondo S. Kanemoto S. Chihara K. (2009). Signalling mediated by the endoplasmic reticulum stress transducer OASIS is involved in bone formation. Nat. Cell Biol. 11, 1205–1211. 10.1038/ncb1963 19767743

[B32] Noske A. B. Costin A. J. Morgan G. P. Marsh B. J. (2008). Expedited approaches to whole cell electron tomography and organelle mark-up *in situ* in high-pressure frozen pancreatic islets. J. Struct. Biol. 161, 298–313. 10.1016/j.jsb.2007.09.015 18069000 PMC2396228

[B33] Paparrizos J. Gravano L. (2015). “k-Shape: Efficient and accurate clustering of time series,” in SIGMOD '15: Proceedings of the 2015 ACM SIGMOD International Conference on Management of Data, Melbourne Victoria Australia, 31 May 2015- 4 June 2015, 1855–1870.

[B48] Raivo Kolde (2019). pheatmap: Pretty Heatmaps. R package version 1.0.12.

[B34] Reimold A. M. Iwakoshi N. N. Manis J. Vallabhajosyula P. Szomolanyi-Tsuda E. Gravallese E. M. (2001). Plasma cell differentiation requires the transcription factor XBP-1. Nature 412, 300–307. 10.1038/35085509 11460154

[B35] Ruggiano A. Foresti O. Carvalho P. (2014). Quality control: ER-associated degradation: Protein quality control and beyond. J. Cell Biol. 204, 869–879. 10.1083/jcb.201312042 24637321 PMC3998802

[B36] Saito A. Hino S. Murakami T. Kanemoto S. Kondo S. Saitoh M. (2009). Regulation of endoplasmic reticulum stress response by a BBF2H7-mediated Sec23a pathway is essential for chondrogenesis. Nat. Cell Biol. 11, 1197–1204. 10.1038/ncb1962 19767744

[B37] Sanchez A. M. Cioffi R. Vigano P. Candiani M. Verde R. Piscitelli F. (2016). Elevated systemic levels of endocannabinoids and related mediators across the menstrual cycle in women with endometriosis. Reprod. Sci. 23, 1071–1079. 10.1177/1933719116630414 26887427

[B46] Sardà-Espinosa A. (2019). Time-series clustering in R using the dtwclust package. R J. 11, 22–43. 10.32614/RJ-2019-023

[B38] Schwabl S. Teis D. (2022). Protein quality control at the Golgi. Curr. Opin. Cell Biol. 75, 102074. 10.1016/j.ceb.2022.02.008 35364487

[B39] Shi J. W. Lai Z. Z. Yang H. L. Yang S. L. Wang C. J. Ao D. (2020). Collagen at the maternal-fetal interface in human pregnancy. Int. J. Biol. Sci. 16, 2220–2234. 10.7150/ijbs.45586 32549767 PMC7294936

[B40] Sun Z. Brodsky J. L. (2019). Protein quality control in the secretory pathway. J. Cell Biol. 218, 3171–3187. 10.1083/jcb.201906047 31537714 PMC6781448

[B41] Symoens S. Malfait F. D'hondt S. Callewaert B. Dheedene A. Steyaert W. (2013). Deficiency for the ER-stress transducer OASIS causes severe recessive osteogenesis imperfecta in humans. Orphanet J. Rare Dis. 8, 154. 10.1186/1750-1172-8-154 24079343 PMC3850743

[B42] Tomoishi S. Fukushima S. Shinohara K. Katada T. Saito K. (2017). CREB3L2-mediated expression of Sec23A/Sec24D is involved in hepatic stellate cell activation through ER-Golgi transport. Sci. Rep. 7, 7992. 10.1038/s41598-017-08703-6 28801610 PMC5554210

[B43] Valetti C. Grossi C. E. Milstein C. Sitia R. (1991). Russell bodies: A general response of secretory cells to synthesis of a mutant immunoglobulin which can neither exit from, nor be degraded in, the endoplasmic reticulum. J. Cell Biol. 115, 983–994. 10.1083/jcb.115.4.983 1955467 PMC2289943

[B44] Van Anken E. Romijn E. P. Maggioni C. Mezghrani A. Sitia R. Braakman I. (2003). Sequential waves of functionally related proteins are expressed when B cells prepare for antibody secretion. Immunity 18, 243–253. 10.1016/s1074-7613(03)00024-4 12594951

[B49] Wickham H (2016). ggplot2: Elegant Graphics for Data Analysis. Springer-Verlag New York. ISBN 978-3-319-24277-4

[B45] Zhang B. (2009). Recent developments in the understanding of the combined deficiency of FV and FVIII. Br. J. Haematol. 145, 15–23. 10.1111/j.1365-2141.2008.07559.x 19183188 PMC2777536

