# Supplementary figures and images for: CREB3L1 and CREB3L2 control Golgi remodelling during decidualization of endometrial stromal cells
Source: Front Cell Dev Biol. 2022 Oct 13;10:986997. doi: 10.3389/fcell.2022.986997 (PMC9608648; doi:10.3389/fcell.2022.986997)

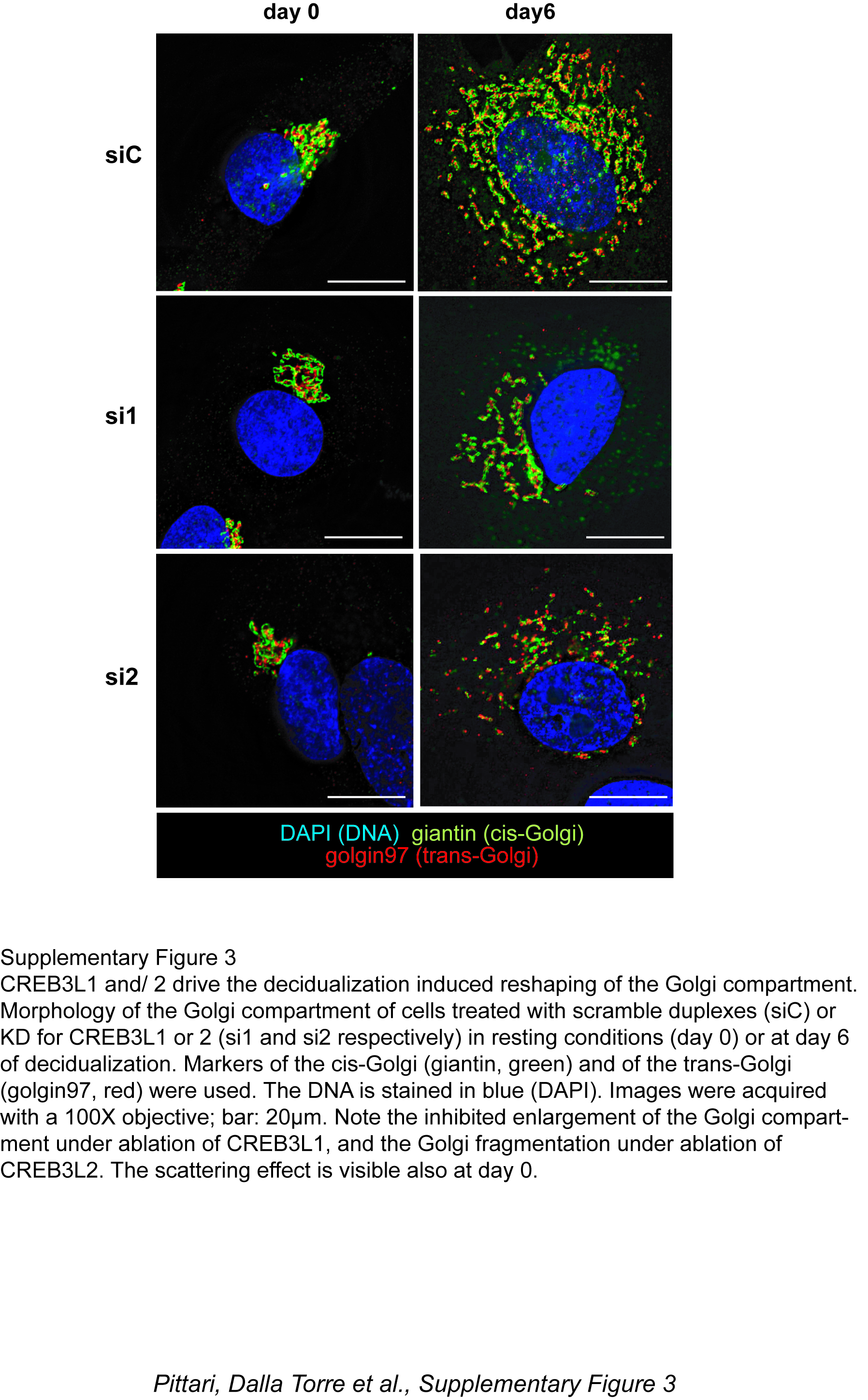

Supplement: Supplementary file 4 [file Image3.TIF]

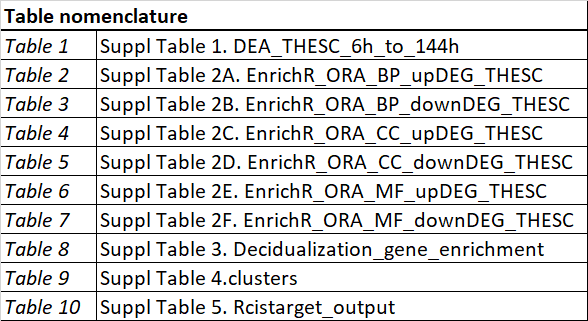

Supplement: Supplementary file 5 [file Image4.TIF]

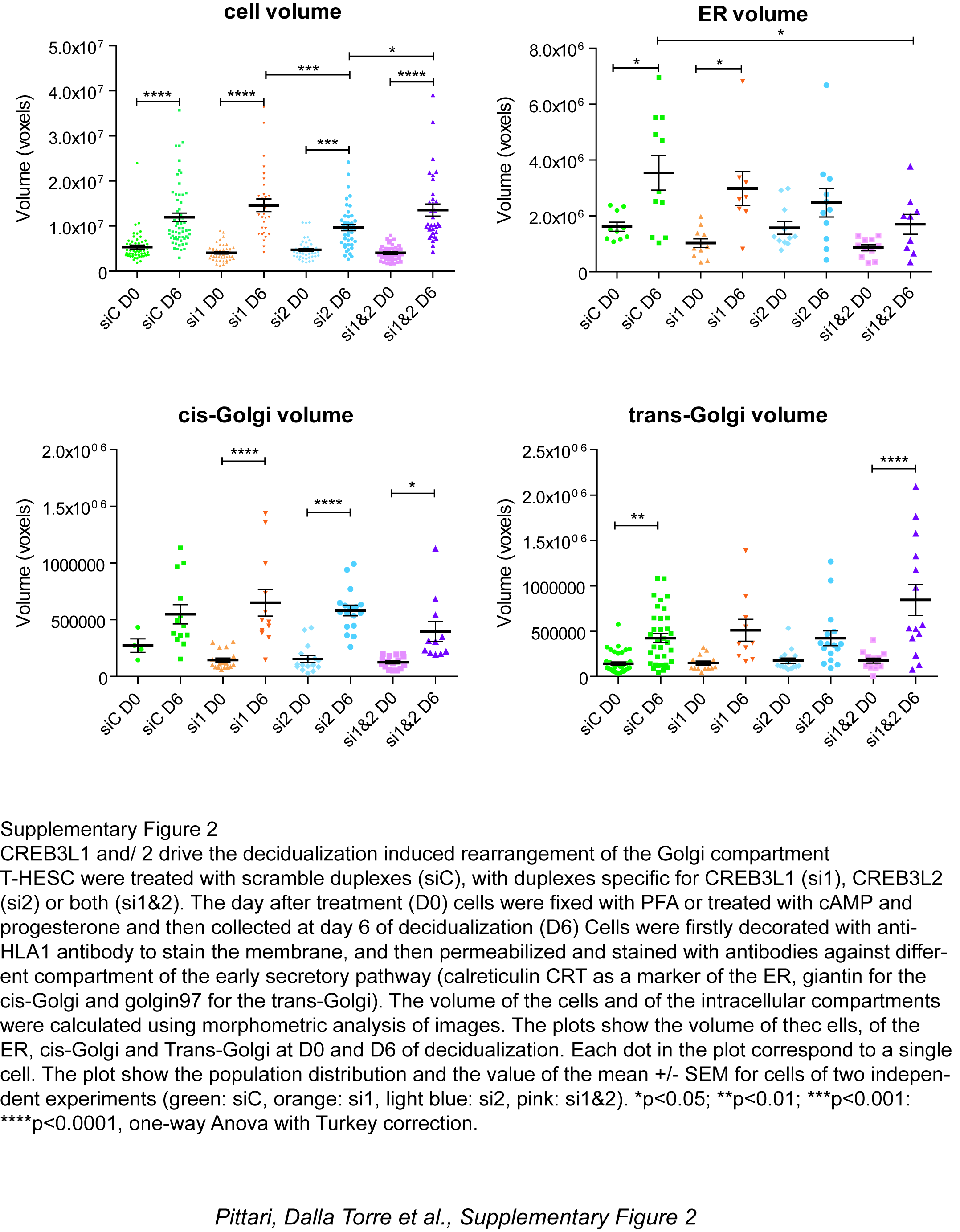

Supplement: Supplementary file 6 [file Image2.TIF]

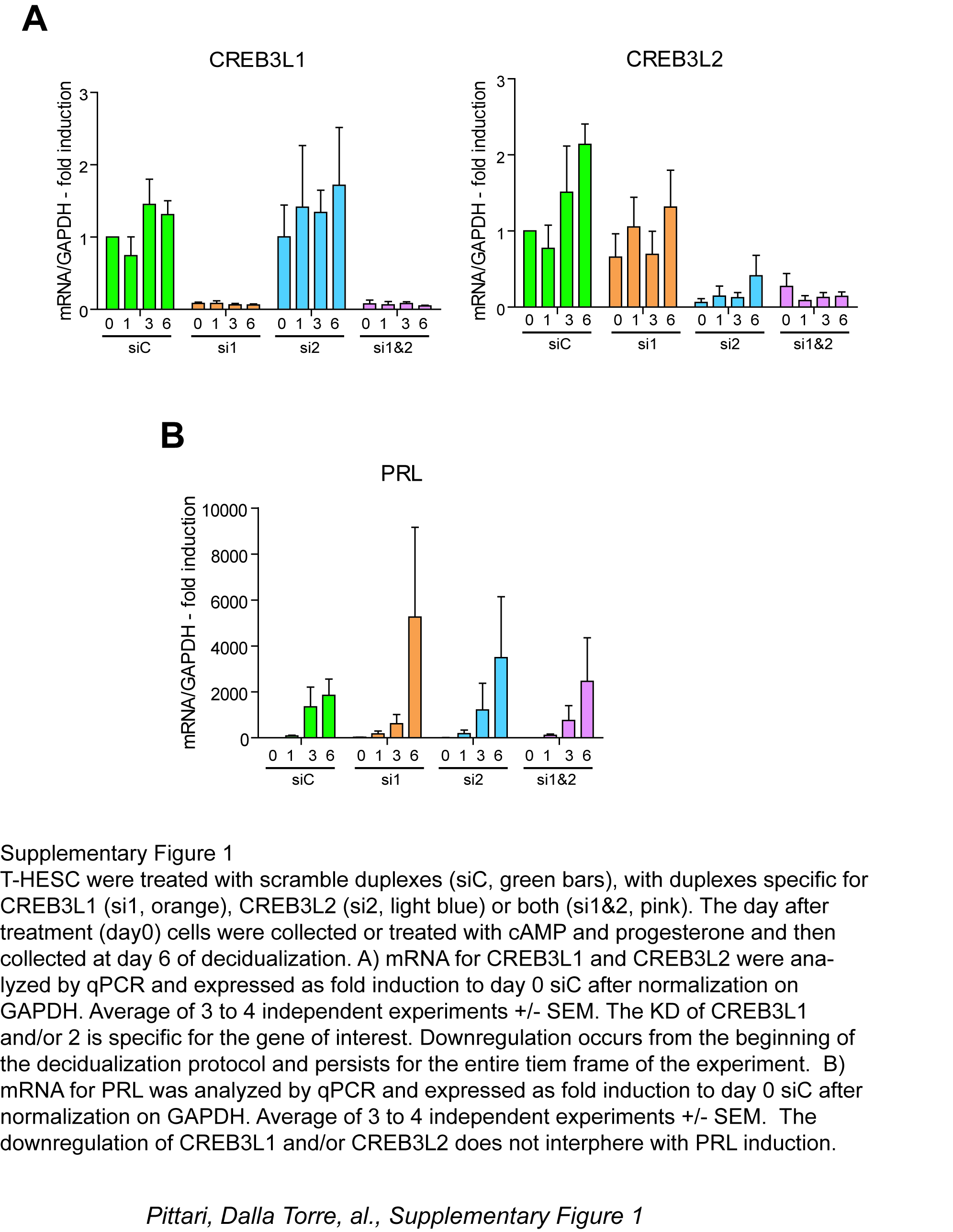

Supplement: Supplementary file 7 [file Image1.TIF]
